# Development of an integrated approach for comparison of in vitro and in vivo responses to particulate matter

**DOI:** 10.1186/s12989-016-0152-6

**Published:** 2016-08-12

**Authors:** Dalibor Breznan, Subramanian Karthikeyan, Marcelle Phaneuf, Prem Kumarathasan, Sabit Cakmak, Michael S. Denison, Jeffrey R. Brook, Renaud Vincent

**Affiliations:** 1 Inhalation Toxicology Laboratory, Hazard Identification Division, Healthy Environments and Consumer Safety Branch, Health Canada, Ottawa, ON Canada; 2 Analytical Biochemistry and Proteomics Laboratory, Mechanistic Studies Division, Healthy Environments and Consumer Safety Branch, Health Canada, Ottawa, ON Canada; 3 Air Health Effects Research, Population Studies Division, Healthy Environments and Consumer Safety Branch, Health Canada, Ottawa, ON Canada; 4 Department of Environmental Toxicology, University of California, Davis, CA USA; 5 Processes Research Section, Air Quality Research Division, Environment and Climate Change Canada, Toronto, ON Canada

**Keywords:** Particulate matter, Cytotoxicity, Inflammation, In vitro, In vivo, Aryl hydrocarbon receptor, Endotoxin, Correlation

## Abstract

**Background:**

Association of particulate matter with adverse health effects has been established in epidemiological studies and animal experiments. Epidemiological studies are difficult to undertake while animal studies are impractical for high-throughput toxicity testing. The ease and rapidity of in vitro tests emphasizes their potential for use in risk assessment of chemicals and particles. We examined the association between in vitro and in vivo responses to ambient particles, to determine the potential of cell-based assays as standalone toxicity screening tools.

**Methods:**

Assays of cytotoxicity and key inflammatory mediators were applied to determine the in vitro biological potency of a panel of urban and mineral particles in J774A.1 macrophages and A549 lung epithelial cells. The particles were also screened for the presence of AhR agonists using the Ah receptor-dependent gene induction assay and for endotoxin using the Limulus amebocyte lysate assay. A subset of the particles with a contrasting in vitro toxicity profile was delivered intratracheally in BALB/c mice to assess their in vivo biological potency. Results from various bioassays were combined within the in vitro and in vivo models. The combined potency measures were examined for associations.

**Results:**

Overall, J774A.1 cells were more sensitive to particle effects than A549 cells. Whereas the combined cytotoxicity estimates were highly correlated between the two cell lines, the combined in vitro inflammatory potency estimates were not, emphasizing functional differences of the two cell types. Secretion of inflammatory markers by J774A.1 cells was correlated with AhR ligand binding profile and endotoxin levels of particles. Particle instillation led to an acute toxicity response in BALB/c mice, with neutrophilia and release of inflammatory mediators. While the combined toxicity estimates were not correlated between in vitro and in vivo models, the combined inflammatory and integrated potency estimates (toxicity and inflammation) approached the threshold for significance (*p* = 0.052) in a correlation within in vitro and in vivo models, with a ranking of fine particle (DWR1), minerals (TiO_2_, CRI) and coarse particles (SRM-, EHC-type) from low to high potency.

**Conclusion:**

Integration of in vitro endpoints shows promise in determining adverse outcomes of particle exposures in vivo. The devised data reduction and computational approach will prove useful in the development of models for assessment of hazard potential of particles; however, distinct models may be needed for particles of different type, such as urban particles vs. mineral particles, nanomaterials.

**Electronic supplementary material:**

The online version of this article (doi:10.1186/s12989-016-0152-6) contains supplementary material, which is available to authorized users.

## Background

Air pollution continues to exert a societal burden by impacting multiple aspects of human health, especially through its association with cardiopulmonary morbidity and mortality [1]. Exposure to airborne particulate matter is epidemiologically associated with increased incidence of cardiopulmonary disease and lung cancer in humans [2, 3]. Moreover, recent studies provide indications that the health impacts of exposure to air pollutants may be more widespread, including epidemiological associations with inflammatory bowel disease and non-specific abdominal pain [4, 5], neuropathologies and cognitive impairment [6, 7], alterations of markers of placental growth and function, and adverse birth outcomes [8, 9], as well as diabetes mellitus [10, 11].

Urban air particles are complex mixtures of chemical and biogenic constituents associated with natural and anthropogenic particle sources and their modification in the atmosphere. Increasing evidence shows that in addition to size, chemical composition of the particles plays an important role in determining their hazard potential. Mitigation of specific constituents of air pollution mixtures and particles with highest hazard potential may present a reasonable regulatory strategy for reduction of adverse population health effects.

Both, in vitro and in vivo approaches are widely utilized for the study of the adverse effects of chemicals and inhaled environmental pollutants by employing individual endpoint assays to determine the adverse effects of particulate materials or to examine specific biological mechanisms of toxicity. Animal studies have been generally accepted as a major approach in the regulatory risk assessment regimes [12–14]. Despite the definitive applicability of in vivo methods to hazard evaluation and risk assessment, issues such as low-throughput, high cost and ethical aspects of animal use hinder their practicality as a toxicity screening tool for large panels of airborne particulates and chemicals. In vitro assays are better suited as screening tools for hazard identification and determination of toxicity, as they are amenable to automation and high-throughput, are cost effective and greatly reduce the dependence on animal use [13, 15, 16]. However, systematic evaluations of the predictive potential of in vitro assays for adverse in vivo impacts of particulate matter are required.

Few attempts have been made to conduct more comprehensive in vitro/in vivo evaluations of panels of particles and particle fractions including gasoline and diesel engine emission organic fractions [17, 18], polyurethane filter-extracted particle suspensions [19], stone particles [20] and nanoparticles [21]. These studies were met with mixed success, whereby the correlations between in vitro and in vivo measurements were poor, with a general lack of rank order of particle hazard potency for organic fractions of gasoline and diesel emissions and for nanoscale particles [17, 18, 21]. In contrast, in vitro inflammatory markers were generally well correlated with in vivo inflammation models of exposure to ambient particles from different emission sources, but not with in vivo respiratory allergy models [19]. Correlations were also observed between in vitro cytokines and in vivo neutrophil counts in lung lavage from rats instilled with mineral stone particles [20]. The lack of consistency between the studies emphasizes the need for further standardization of in vitro approaches, including variables such as culture conditions, time course, choice of a wide range of bioassays, particle types, and the validation against relevant in vivo effects.

The usefulness of in vitro studies is highlighted by demonstrations of coherence of the particle-induced biological effects in cultured cells with in vivo responses from a controlled human exposure study [22] and with concomitant epidemiological observations of associations of Utah Valley hospital respiratory admissions and PM_10_ emissions from a local steel mill source [23]. Particles extracted from filters collected from the Utah Valley before, during, and after a temporary closure of a steel mill differentially induced neutrophilia and IL-8 production in human subjects instilled with the particles via bronchoscopy. Interleukin-8 production was also stimulated in human airway epithelial cells exposed to the same particles in a corresponding manner, with a comparable potency profile [22].

In the present study, we assessed the toxicity and inflammatory potential of a panel of airborne particulates and reference particles in cell lines in vitro and in BALB/c mice exposed in vivo to particles at 24 h post-intratracheal instillation. An approach was developed to summarize large datasets to facilitate a comparison between the in vitro and in vivo responses to particles and to evaluate the level of concordance between the two models for toxicity testing.

## Methods

### Particulate materials

The particulate preparations SRM-1648 (St. Louis total suspended particles, TSP; urban particulate matter reference material), SRM-1649a (Washington TSP; organics), SRM-1879 (cristobalite, SiO_2_ reference material) and SRM-154b (fine titanium dioxide; TiO_2_ reference material) were obtained from the National Institute of Standards and Technology (Gaithersberg, MD, USA). Cristobalite particles will be referred to as CRI throughout the manuscript. The TiO_2_ particles were washed in methanol and phosphate buffered saline (PBS) to remove potential naphthalene contaminant [24]. The PM_2.5_ DWR1 particles were collected in Toronto as described before [24]. The urban TSP preparations EHC-93, EHC-98 and EHC-2000 refer to materials collected in Ottawa as described before [25]. Particulate materials were weighed and resuspended in sterile particle preparation solution (Tween-80, 25 μg/mL; NaCl, 0.19 % w/v) to a concentration of 10 mg/mL using a Dounce glass homogenizer. After vortexing, the particle suspensions were sonicated in ice-cold water for 20 min and homogenized with 25 full strokes of the homogenizer piston. The particle stocks were aliquoted into sterile centrifuge tubes with O-ring seals and sterilized in a water bath at 56 °C for 30 min. These were kept frozen at −80 °C, until use [26]. All materials were analyzed for endotoxin using the chromogenic Limulus amebocyte lysate assay (Lonza, Walkersville, MD, USA).

The materials EHC-93, EHC-98, EHC-2000 (Ottawa), SRM-1648 (St. Louis) and SRM-1649 (Washington) are TSP matter from urban environments, characterized by enrichment in elements including aluminium (20–30 mg/g), calcium (26–128 mg/g), iron (16–30 mg/g), magnesium (9–16 mg/g), lead (5–14 mg/g), titanium (2–3 mg/g) and zinc (2–11 mg/g), in the milligram per gram range. DWR1 is a PM_2.5_, with 10–100-fold less components from crustal origin, e.g. aluminium (0.8 mg/g), iron (1 mg/g), magnesium (1.4 mg/g), lead (0.05 mg/g), titanium (0.06 mg/g). SiO_2_ and TiO_2_ are mineral particles containing a variety of trace elements. Available physicochemical information from in-house analysis and NIST Certificates of Analysis are presented in supplemental information (Additional file 1: Table S1).

### Cell culture

A549 human alveolar type II epithelial (Caucasian male, lung carcinoma) and J774A.1 murine monocyte/macrophages (BALB/c female, cell sarcoma) cell lines were from ATCC (Manassas, VA, USA). H1L1.1c2 murine hepatoma cells containing a stably transfected Ah receptor-dependent luciferase reporter gene were described elsewhere [27]. A549 were cultured in M199 with 2 mM L-glutamine and 10 % FBS (Invitrogen-Gibco, Burlington, ON, Canada). J774A.1 were cultured in DMEM medium (Thermo-Fisher, Nepean, ON, Canada) with 2 mM L-glutamine, 4.5 g/L glucose and 10 % FBS. H1L1.1c2 cells were cultured in MEM Alpha medium (Thermo-Fisher) supplemented with 10 % FBS and 2 mM L-glutamine. For all cytotoxicity assays except BrdU incorporation, cells were seeded in 96-well (0.3 cm^2^ well) plates at cell densities of 4 × 10^4^ (A549) and 8 × 10^4^ cells/cm^2^ (J774A.1). For BrdU incorporation, cells were seeded at 1 × 10^4^ (A549) and 2 × 10^4^ cells/cm^2^ (J774A.1). For the LDH assay, A549 and J774A.1 cells were cultured in serum-free media supplemented with 2 mM L-glutamine, 4.5 g/L glucose (J774A.1 cells only) and 6 mg/mL BSA. H1L1.1c2 cells were seeded in 24-well (0.5 cm^2^ well) plates at a density of 1 × 10^4^ cells per well, in 1 mL of complete medium. The cells were then incubated for 24 h at 37 °C in 5 % CO_2_ and 100 % relative humidity for attachment.

### In vitro exposure to particles

Particle suspensions were thawed to room temperature and sonicated for 20 min in ice-cold, ultrasonic water bath, and diluted (1–2 mg/mL) in complete media devoid of serum for dosing cells. The diluted particle suspensions were sonicated for 5 min in an ice-cold, ultrasonic water bath prior to dosing the cells. A549, J774A.1 and H1L1.1c2 cell monolayers were dosed with a 100 μL suspension of particles resulting in 0, 10, 20, 40, 80 and 100 μg/well (0, 30, 60, 120, 240 and 480 μg/cm^2^) in a final volume of 200 μL/well, 5 % FBS. The A549 and J774A.1 cell culture plates were incubated at 37 °C for 24 h before the cytotoxicity measurements were carried out. Culture plates containing H1L1.1c2 cells were incubated at 37 °C for 4 h before they were assayed for induction of aryl hydrocarbon response.

### Cytotoxicity assessment

Following 24 h incubation with particles, A549 and J774A.1 cells were assayed for cytotoxicity using resazurin reduction assay (Alamar Blue), ATP assay, BrdU incorporation assay and lactate dehydrogenase (LDH) assay, and in H1L1.1c2 cells for Ah receptor-dependent gene induction, as briefly outlined below. Assays were performed using aliquots from cell culture supernatants and lysates that were clarified by centrifugation to remove the remnants of particles that could potentially interfere with the assay reagent chemistry and detection methods. The approach utilized has been previously described [28]. ATP assay was performed by top reading.

#### Resazurin reduction assay

The spent medium was collected and replaced with 100 μL of fresh complete medium containing a 1:10 dilution of Resazurin (Alamar Blue) reagent (Biosource, Camarillo, CA, USA) and fluorescence was measured at λEx 530/25 nm and λEm 590/35 nm using Cytofluor 2350 fluorometer [28], 5 min and 2 h after incubation at 37 °C, 5 % CO_2_). Rate of reduction is the delta fluorescence at 2 h – 5 min. Five independent experiments were conducted (*n* = 5), with triplicate wells within each experiment.

#### ATP assay

Cellular ATP content was assessed using the ViaLight Plus assay (Lonza, Walkersville, MD, USA). Cells were lysed with 10 μL of the assay cell lysis reagent for 10 min, followed by 25 μL of ATP-monitoring reagent (AMR) containing firefly luciferase, luciferin, pyrophosphate, BSA and magnesium ions. After 2 min incubation in the dark, the cellular ATP content was determined by top reading luminescence measurements using LMax luminometer. Luminescence (rate of photon emission) is directly proportional to concentration of ATP substrates). The ATP assay was performed in three independent experiments (*n* = 3), with triplicate wells within each experiment.

#### BrdU incorporation

Following exposure to particles, 5-bromo-2-deoxyuridine (BrdU) was added to each well at 100 μM and the cells were returned to the incubator for 2 h. The culture medium was removed by inversion of the 96-well plates, and the cells were fixed for 30 min at room temperature using the FixDenat solution (Roche Diagnostics, Indianapolis, IN, USA). The cells were washed with blocking buffer (1 % BSA in PBS), followed by an incubation with 100 μL of anti-BrdU antibody conjugated with peroxidase (anti-BrdU-POD, Fab fragments) for 90 min at room temperature. After extensive washing, cells were incubated with 100 μL of the 3,3’,5,5’-tetramethylbenzidine (TMB) chromogenic substrate for 10 min at room temperature in the dark, and the reaction product was quantified at 370 nm using SpectraMax Plus spectrophotometer. Three independent assays (*n* = 3), with triplicate wells within each assay were conducted.

#### LDH assay

Culture supernatants were recovered and the cells were lysed by a rapid freeze-thaw cycle. LDH activity was measured in the clarified supernatants and the cell lysates by spectrophotometry at 450 nm (SpectraMax Plus) from production of NADH from lactate coupled with the instantaneous conversion of tetrazolium to a red formazan by NADH diaphorase (CytoTox 96 assay, Promega, Madison, WI, USA). Release of cellular LDH was determined from activity in supernatant over total LDH (supernatant plus lysate). Three (A549, *n* = 3) or four (J774A.1, *n* = 4) independent assays, with triplicate wells within each experiment were conducted.

#### Assay of Ah receptor-dependent gene induction

After dosing with particles, H1L1.1c2 cell plates were incubated at 37 °C for 4 h, followed by two washes in ice-cold PBS, and 15 min lysis on ice using 50 μL/well of luciferase assay system (Promega Corporation) lysis buffer, on a plate shaker (250 rpm). The lysates were transferred to v-bottom plates and centrifuged at 3000 × g (2 min, 4 °C) using a Sorvall Legend RT centrifuge. Clarified cell lysates (25 μL) were transferred into a white, clear-bottomed 96-well plate, 50 μL of luciferase substrate was added by injection using an L-Max luminometer, followed by 15 s integrated luminescence reading per well. Three independent experiments were conducted (*n* = 3), with triplicates within each experiment.

### Cytokine secretion in vitro

Cell culture supernatants of A549 cells were assessed for Interleukin (IL)-1β (0.13 ± 0.01 pg/mL basal level in control cells ± standard error mean), IL-1ra (n.d.; not detected), IL-2 (n.d.), IL-4 (n.d.), IL-5 (n.d.), IL-6 (430 ± 111), IL-7 (n.d.), IL-8 (200 ± 4), IL-9 (n.d.), IL-10 (12.5 ± 1.9), IL-12 (p70) (n.d.), IL-13 (n.d.), IL-15 (n.d.), IL-17 (n.d.), basic Fibroblast Growth Factor (FGF) (n.d.), Eotaxin (n.d.), Granulocyte Colony-Stimulating Factor (G-CSF) (n.d.), Granulocyte Macrophage Colony-Stimulating Factor (GM-CSF) (2.8 ± 0.4), Interferon (IFN)-γ (n.d.), IFN-γ Inducible Protein (IP)-10 (n.d.), Monocyte/Macrophage Chemoattractant Protein (MCP)-1 (701.5 ± 44.8), Macrophage Inflammatory Protein (MIP)-1α (n.d.), MIP-1β (0.9 ± 0.5), Platelet-Derived Growth Factor (PDGF)-BB (n.d.), Regulated on Activation, Normal T cell Expressed and Secreted (RANTES) (n.d.), Tumor Necrosis Factor (TNF)-α (0.1 ± 0.1), and Vascular Endothelial Growth Factor (VEGF) (n.d.), while cell culture supernatants of J774A.1 were assessed for IL-1α (1.2 ± 0.1), IL-1β (5.9 ± 1.8), IL-2 (n.d.), IL-3 (n.d.), IL-4 (n.d.), IL-5 (n.d.), IL-6 (9.2 ± 1.0), IL-9 (n.d.), IL-10 (12.5 ± 1.9), IL-12 (p40) (n.d.), IL-12 (p70) (n.d.), IL-13 (n.d.), IL-17A (n.d.), Eotaxin (n.d.), G-CSF (n.d.), GM-CSF (9.4 ± 1.3), IFN-γ (n.d.), Keratinocyte Chemoattractant (KC) (n.d.), MCP-1 (n.d.), MIP-1α (above assay limit), MIP-1β (n.d.) and RANTES (239 ± 28) using Bio-Plex cytokine panels (Bio-Rad Laboratories, Mississauga, ON, Canada), with a Bio-Plex 200 array system.

### In vivo exposures to particles

Seven weeks-old, pathogen-free BALB/c male mice (~25 g) were from Charles-River (Saint-Constant, QC, Canada). Mice were housed in shoe-box plexiglass cages, on woodchip bedding, provided with mouse chow and water ad libitum, maintained under HEPA air filtration at 22 +/− 2 °C, ~50 % humidity and were held to a 12-h light/dark cycle. The animals were acclimatized for at least 1 week prior to experimentation. The animal treatment protocol was reviewed and approved by the Animal Care Committee of Health Canada. The particle preparations were thawed at room temperature, sonicated for 10 min, diluted in saline and instilled intratracheally (50 μL volume) using the MicroSprayer aerosolizer (Penn-Century, Philadelphia, PA, USA) at doses of 0, 50, 100, 250 μg into anaesthetized mice in supine position on an angled stand (*n* = 5 animals per dose). Mice were euthanized after 24 h, bronchioalveolar lavage (BAL) fluid and blood plasma were obtained as described before [25]. BAL cells were counted and differential cytology (Wright-Giems stain) was determined on CytoSpin preparations (Thermo Shandon, Pittsburgh PA, USA) prepared on glass slides using the Cytospin 3 centrifuge. Cell differential counts of alveolar macrophages, neutrophils, lymphocytes, monocytes, eosinophils, basophils and band cells (immature neutrophils) were determined from a pair of slides for each animal. Fold change (i.e. treatment/control) of the cell counts are presented. The values are based on the percentage of cell type in a differential profile multiplied by the total cell number (calculated as cell # per ml BAL × volume of instilled lavage fluid in the animal) to obtain an estimate of the absolute number of the cell types recovered from lavage.

### Biochemical assays in vivo

8-Isoprostaglandin-2a (Isoprostane) was quantitated from blood plasma samples by competitive Enzyme Immuno-Assay (EIA) following manufacturer-recommended procedures (Cayman Chemical, Ann Arbor, MI, USA). LDH in BAL was analyzed essentially as described for cell cultures. Total protein was measured by the Coomassie Plus (Bradford) assay as per manufacturer’s protocol (Pierce, Rockford, IL, USA).

### Cytokine secretion in vivo

The profile of IL-1α (0.1 ± 0.1 pg/mL basal level in control cells ± standard error mean), IL-1β (1.3 ± 0.2), IL-4 (n.d.; not detected), IL-5 (0.5 ± 0.5), IL-6 (0.1 ± 0), IL-10 (0.3 ± 0.13), GM-CSF (1.3 ± 0), KC ((3.8 ± 0.9), MIP-1α (25.4 ± 8.8), RANTES (0.3 ± 0.2) and TNF-α (3.7 ± 1.0) was determined by Bio-Plex analyses of BAL samples using Bio-Plex cytokine panels, with a Bio-Plex 200 array system. Basal levels in control cells are in parentheses above and represent pg/mL ± standard error mean measured in cell culture supernatants and n.d. denotes cytokine/chemokine not detected.

### Particle potency estimates

The dose–response data for cell viability, cytokine secretion and Ah receptor-dependent gene induction (AhR response was scaled by dividing 20-fold to relate its magnitude to the other endpoints), were normalized within an experiment for all doses (including 0 μg dose control), to the grand mean value of all controls (0 μg dose of particles), to obtain fold-effect (FE) for each particle dose. Potency (β) was derived from

$$ \mathrm{F}\mathrm{E} = {\left(\mathrm{Dose} + 1\right)}^{\upbeta} $$

where β is the rate of change of dose with respect to the logarithm of fold-effect for a given endpoint [24]. The dose–response data were fitted using CurveExpert v1.3 (D. Hyams, Hixson, TN, USA).

The following potencies were calculated: potency for viability (β_V_) in a given assay, potency for Ah receptor-dependent gene induction (β_AhR_), and potencies for secretion of a cytokine (β_I_). β_I_ was adjusted for cell viability according to

$$ {\upbeta}_{\mathrm{I}-\mathrm{V}} = {\upbeta}_{\mathrm{I}}-{\upbeta}_{\mathrm{V}} $$

where β_I-V_ represents an unbiased potency estimate that allows distinction of apparent down-regulation of a cytokine from the absence of cytokine secretion by dead cells, or an apparent up-regulation of cytokine expression in cells simply due to an increase of cell mass in the wells.

The potencies were combined as follows;

1. An average cytotoxic potency in each cell line was calculated using assay specific potencies according to:

$$ \begin{array}{l}{\upbeta_{\mathrm{V}}}_{\mathrm{A}549} = \mathrm{A}\mathrm{verage}\ \left({\upbeta}_{\mathrm{V}\ \mathrm{L}\mathrm{D}\mathrm{H}\ \mathrm{A}549},\;{\upbeta}_{\mathrm{V}\ \mathrm{A}\mathrm{B}\ \mathrm{A}549},\;{\upbeta}_{\mathrm{V}\ \mathrm{A}\mathrm{T}\mathrm{P}\ \mathrm{A}549},\;{\upbeta}_{\mathrm{V}\ \mathrm{B}\mathrm{RDU}\ \mathrm{A}549}\right)\\ {}{\upbeta}_{\mathrm{V}\ \mathrm{J}774\mathrm{A}.1} = \mathrm{A}\mathrm{verage}\ \left({\upbeta}_{\mathrm{V}\ \mathrm{L}\mathrm{D}\mathrm{H}\ \mathrm{J}774\mathrm{A}.1},\;{\upbeta}_{\mathrm{V}\ \mathrm{A}\mathrm{B}\ \mathrm{J}774\mathrm{A}.1},\;{\upbeta}_{\mathrm{V}\ \mathrm{A}\mathrm{T}\mathrm{P}\ \mathrm{J}774\mathrm{A}.1},\;{\upbeta}_{\mathrm{V}\ \mathrm{B}\mathrm{RDU}\ \mathrm{J}774\mathrm{A}.1}\right)\\ {}{\upbeta}_{\mathrm{V}\ \mathrm{CELLS}} = \mathrm{A}\mathrm{verage}\ \left({\upbeta}_{\mathrm{V}\ \mathrm{A}549},\;{\upbeta}_{\mathrm{V}\ \mathrm{J}774\mathrm{A}.1}\right)\end{array} $$

2) Biological reactivity (β_R_) of the particles was defined as any deviation from baseline in cytotoxicity assays. β_R__CELLS_ was calculated by averaging the absolute values (to capture any deviation from baseline control levels) of the β_V_ according to:

$$ \begin{array}{l}{\upbeta_{\mathrm{R}}}_{\mathrm{A}549} = \mathrm{A}\mathrm{V}\mathrm{G}\ \left(\left|{\upbeta_{\mathrm{V}}}_{\mathrm{LDH}\ \mathrm{A}549}\left|,\ \right|{\upbeta_{\mathrm{V}}}_{\mathrm{A}\mathrm{B}\ \mathrm{A}549}\left|,\ \right|{\upbeta_{\mathrm{V}}}_{\mathrm{A}\mathrm{TP}\ \mathrm{A}549}\left|,\ \right|{\upbeta_{\mathrm{V}}}_{\mathrm{BRDU}\ \mathrm{A}549}\right|\right)\\ {}{\upbeta_{\mathrm{R}}}_{\mathrm{J}774\mathrm{A}.1} = \mathrm{A}\mathrm{V}\mathrm{G}\ \left(\left|{\upbeta_{\mathrm{V}}}_{\mathrm{LDH}\ \mathrm{J}774\mathrm{A}.\mathrm{A}.1}\left|,\ \right|{\upbeta_{\mathrm{V}}}_{\mathrm{A}\mathrm{B}\ \mathrm{J}774\mathrm{A}.1}\left|,\ \right|{\upbeta_{\mathrm{V}}}_{\mathrm{A}\mathrm{TP}\ \mathrm{J}774\mathrm{A}.1}\left|,\ \right|{\upbeta_{\mathrm{V}}}_{\mathrm{BRDU}\ \mathrm{J}774\mathrm{A}.1}\right|\right)\\ {}{\upbeta}_{\mathrm{R}\ \mathrm{CELLS}} = \mathrm{A}\mathrm{verage}\ \left({\upbeta}_{\mathrm{R}\ \mathrm{A}549},\;{\upbeta}_{\mathrm{R}\ \mathrm{J}774\mathrm{A}.1}\right)\end{array} $$

3) An average potency for inflammation was calculated within each cell line using +1*β_I-V_for chemokines and pro-inflammatory cytokines and −1*β_I-V_ for anti-inflammatory cytokines. Two different estimates of inflammatory potency were calculated.
  - β_I-V__HI_, an average considering the anti-inflammatory effect of IL-10 (−1*β_I-V_ for IL10).
  - β_I-V__LO_, an average considering anti-inflammatory effects of IL-6 and IL-10.

4) β_R__CELLS_ and one of the two estimates of β_I-V_ were further integrated to calculate cell-specific Integrated Beta potencies (Iβ). For example,

$$ \begin{array}{l}{\mathrm{I}\upbeta}_{\mathrm{A}549\ \mathrm{H}\mathrm{I}} = \mathrm{A}\mathrm{verage}\ \left({\upbeta}_{\mathrm{R}\ \mathrm{A}549},{\upbeta}_{\mathrm{I}-\mathrm{V}\ \mathrm{A}549\ \mathrm{H}\mathrm{I}}\right)\\ {}{\mathrm{I}\upbeta}_{\mathrm{J}774\mathrm{A}.1\ \mathrm{L}\mathrm{O}} = \mathrm{A}\mathrm{verage}\ \left({\upbeta}_{\mathrm{R}\ \mathrm{J}774\mathrm{A}.1},{\upbeta_{\mathrm{I}-\mathrm{V}}}_{\mathrm{J}774\mathrm{A}.1\ \mathrm{L}\mathrm{O}}\right)\end{array} $$

5) β_R_ and β_I-V_ were integrated across the cell lines as follows.

$$ \begin{array}{l}{\upbeta_{\mathrm{R}}}_{\mathrm{CELLS}} = \mathrm{A}\mathrm{verage}\ \left({\upbeta}_{\mathrm{R}\ \mathrm{A}549},{\upbeta}_{\mathrm{R}\ \mathrm{J}774\mathrm{A}.1},{\upbeta}_{\mathrm{AhR}/20}\right)\\ {}{\upbeta}_{\mathrm{I}-\mathrm{V}\ \mathrm{H}\mathrm{I}} = \mathrm{A}\mathrm{verage}\ \left({\upbeta}_{\mathrm{I}-\mathrm{V}\ \mathrm{A}549\ \mathrm{H}\mathrm{I}},{\upbeta}_{\mathrm{I}-\mathrm{V}\ \mathrm{J}774\mathrm{A}.1\ \mathrm{H}\mathrm{I}}\right)\\ {}{\upbeta}_{\mathrm{I}-\mathrm{V}\ \mathrm{L}\mathrm{O}} = \mathrm{A}\mathrm{verage}\ \left({\upbeta}_{\mathrm{I}-\mathrm{V}\ \mathrm{A}549\ \mathrm{L}\mathrm{O}},{\upbeta}_{\mathrm{I}-\mathrm{V}\ \mathrm{J}774\mathrm{A}.1\ \mathrm{L}\mathrm{O}}\right)\\ {}{\upbeta}_{\mathrm{I}-\mathrm{V}\ \mathrm{CELLS}} = \mathrm{A}\mathrm{verage}\ \left({\upbeta}_{\mathrm{I}-\mathrm{V}\ \mathrm{H}\mathrm{I}},{\upbeta}_{\mathrm{I}-\mathrm{V}\ \mathrm{L}\mathrm{O}}\right)\end{array} $$

6) β_R_ and one of the two estimates of β_I-V_ across cell lines were further integrated to a grand Integrated potency (Iβ). For example,

$$ \begin{array}{l}{\mathrm{I}\upbeta}_{\mathrm{HI}} = \mathrm{Average}\ \left({\upbeta}_{\mathrm{R}},\;{\upbeta}_{\mathrm{I}-\mathrm{V}\ \mathrm{H}\mathrm{I}}\right)\\ {}{\mathrm{I}\upbeta}_{\mathrm{LO}} = \mathrm{Average}\ \left({\upbeta}_{\mathrm{R}},\;{\upbeta}_{\mathrm{I}-\mathrm{V}\ \mathrm{L}\mathrm{O}}\right)\\ {}\mathrm{I}\upbeta = \mathrm{Average}\ \left({\mathrm{I}\upbeta}_{\mathrm{HI}},\ {\mathrm{I}\upbeta}_{\mathrm{LO}}\right)\end{array} $$

In a similar fashion, potency estimates were determined for each in vivo endpoint. A potency estimate for acute lung toxicity, β_T BALB/c_ was determined from the average of potencies for BAL endpoints (protein, LDH, 8-isoprostane, neutrophils, macrophages, band cells, lymphocytes), while a potency estimate for inflammation, β_I__BALB/c_ was determined from the potencies of particles for inducing the release of cytokines and chemokines. As described for the in vitro inflammatory potency estimates, β_I HI_, an average considering the anti-inflammatory effect of IL-10 (−1*β_I_ for IL10) and β_I LO_, an average considering anti-inflammatory effects of IL-6 and IL-10 were determined. An integrated potency estimate that incorporates all in vivo data, Iβ _BALB/c_ was obtained from the average of β_T__BALB/c_ and β_I BALB/c_.

### Statistical analyses

To ensure that assays were sensitive to the dose of particles, fold effect (FE) data were first analyzed by two-way ANOVA with *DOSE* (0, 10, 20, 40, 80, 160 μg/well) for in vitro data, or (0, 50, 100, 250 μg) for in vivo data, and *Particulate Matter (PM)* as factors. Datasets not meeting normality and equal variance were subjected to log_10_, inverse or square root transformations (in the order given) until the assumptions were met, or else rank transformed prior to analyses. Pairwise multiple comparisons were carried out using Tukey’s procedure to elucidate the pattern of significant effects (α = 0.05). The analyses were conducted using SigmaPlot, version 12.5 (Systat Software, Inc., San Jose, CA, USA).

Hierarchical clustering of the pattern of cytokine secretion by A549 and J774 in response to particle exposure were conducted using the GenePattern webtool (http://www.broadinstitute.org/cancer/software/genepattern) [29], and visualized as heatmaps using Java TreeView plugin version 1.16.r2 (http://jtreeview.sourceforge.net) [30].

Linear regression between corresponding individual or combined potency estimates in vitro and in vivo was conducted using Sigmaplot v12.5 and depicted using Microsoft Excel 2010 (Microsoft Corp., Redmond, WA, USA). The strength of the relationship between every two variables was described by a correlation coefficient R and the significance of the hypothesis test by the p-value of 0.05 (two-tailed test), or one-tailed test, where applicable (i.e. consistent directionality of the variables).

The correlations presented are performed between in vitro and in vivo matched endpoints across all particles, based on individual particle potencies (Table 1) or combined potency estimates (average of endpoints) for toxicity, inflammation, or integrated inflammation plus toxicity (Tables 2, 3, and 5) all eight particles tested in vitro (EHC-93, EHC-98, EHC-2000, SRM-1648, SRM-1649, DWR1, TiO_2_, SiO_2_) or for five particles tested in vivo (EHC-2000, SRM-1649, DWR1, TiO_2_, SiO_2_) for Table 5.

**Table 1:** Pearson correlations for cytotoxic potency and cytokine inductions in cell lines exposed to particles

J774A.1 vs. A549 cells	Assay				Cytokine				
	AB	ATP	BrdU	LDH_c_	GM-CSF	IL-1β	IL-6	IL-10	TNF-α
R	0.478	0.703	**0.792**	0.455	0.071	−0.134	−0.051	0.446	−0.192
*p* (2-tailed)	*0.231*	*0.052*	***0.019***	*0.257*	*0.867*	*0.752*	*0.905*	*0.268*	*0.649*

Numbers in bold represent statistically significant correlations (*p* ≤ 0.05)

Correlations of corresponding individual endpoints across the cell lines were conducted

**Table 2:** Pearson correlations for the combined in vitro potency estimates of cell lines exposed to particles

J774A.1 vs. A549 cells	Cytotoxicity		Cytokine induction		
	β_V_	β_R_	β_I-V_	β_I-V LO_	β_I-V HI_
R	**0.741**	**0.729**	0.020	−0.089	−0.108
*p* (2-tailed)	***0.035***	***0.040***	*0.963*	*0.835*	*0.800*

Consensus cytotoxic potency β_V_ is calculated from in vitro assays conducted in individual cell lines

Biological reactivity of the particles β_R_ represent absolute values of cytotoxic potency

Consensus inflammatory potency β_I-V_ represents the average unbiased particle potency response for inducing a cytokine response, where LO and HI are the lower and upper estimates of the index based on the pro- and anti-inflammatory effects of some cytokines

Numbers in bold represent statistically significant correlations (*p* ≤ 0.05)

**Table 3:** Pearson correlations of cytokine induction in J774A.1 and A549 cells versus AhR response in H1L1.1c2 cells upon exposures to particles

Cytokine induction (J774A.1/A549) vs AhR response	J774A.1							A549							
	GM-CSF	IL-1α	IL-1β	IL-6	IL-10	RANTES	TNF-α	GM-CSF	IL-1β	IL-6	IL-8	IL-10	MCP-1	MIP-1β	TNF-α
R	0.699	0.645	**0.718**	**0.867**	−0.520	0.697	**0.822**	0.009	−0.517	−0.271	−0.484	−0.629	−0.090	−0.652	−0.523
*p* (2-tailed)	*0.054*	*0.084*	***0.045***	***0.005***	*0.186*	*0.055*	***0.012***	*0.982*	*0.190*	*0.516*	*0.224*	*0.095*	*0.832*	*0.080*	*0.183*

Numbers in bold represent statistically significant correlations (*p* ≤ 0.05)

In addition, a selection of key potency estimates were ranked and the strength of their relationship was reassessed using Spearman’s rank-order correlation test (one-tailed test), as the selected variables were observed to have a consistent positive relationship, with a significance level of 0.05, using Spearman’s Rho calculator (http://www.socscistatistics.com/tests/spearman/default2.aspx; Social Science Statistics).

## Results

### Cytotoxicity and Ah receptor-dependent gene induction

**Fig. 1:**
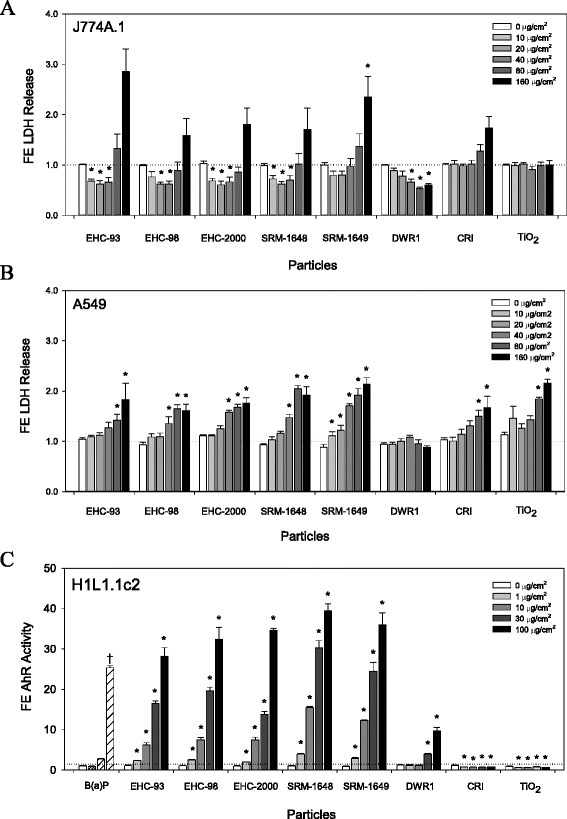
Lactate Dehydrogenase (LDH) release into cell culture supernatants of J774A.1 (**a**) and A549 (**b**) cells exposed for 24 h to particles, and Aryl hydrocarbon receptor (AhR) induction by particles in the H1L1.1c2 cells after a 4 h exposure (**c**). For the LDH assay, data represent LDH release, adjusted for total cellular LDH content. For the AhR assay, B[a]P was used as a positive control (0, 1, 10, 100 nM). All values are presented as average fold-effect (FE) over control ± standard error (J774A.1, *n* = 4; A549, *n* = 3; H1L1.1c2, *n* = 3). Two way ANOVA; J774A.1, PM × Dose interaction, *p* < 0.001, asterisks (*) represent effects significantly different from Dose 0 control, Tukey test, *p* < 0.05; A549, PM × Dose interaction, *p* < 0.001, asterisks (*) represent effects significantly different from Dose 0 control, Tukey test, *p* < 0.05. Two way ANOVA; AhR, PM × Dose interaction, *p* < 0.001, asterisks (*) represent effects significantly different from Dose 0 control, Tukey test, *p* < 0.05; one way ANOVA; B(a)P, Concentration, *p* = 0.016, Conc. 100 vs. 1 (†), Tukey test, *p* < 0.05

Exposures of A549 and J774A.1 cells to particles revealed cytotoxicity manifested by increased release of LDH into cell culture supernatants (Fig. 1a and b) which was particle type- and dose-dependent (two way ANOVA, PM × Dose, *p* < 0.001), most prominent at the highest doses of the particles. The LDH assay also revealed a significant decrease in LDH release in J774A.1 cells exposed to lower and intermediate doses of urban particles in comparison to baseline levels, but not in response to mineral particles (Fig. 1a). This response was not observed in A549 cells exposed to particles (Fig. 1b). Furthermore, we observed reduced metabolic activity manifested by decreased resazurin reduction (Additional file 2: Figure S1A and S1B), cellular ATP levels (Additional file 2: Figure S1C and S1D) as well as attenuated cell proliferation measured by BrdU incorporation assay (Additional file 2: Figure S1E and S1F). The presence of Ah receptor agonists was detected in urban particles, but not mineral particles (two way ANOVA, PM × Dose, *p* < 0.001) measured by AhR-mediated luciferase reporter gene activity in H1L1.1c2 cells. A small but significant decrease in AhR-mediated gene activity below baseline levels was detected in cells exposed to the mineral particles (Fig. 1c).

**Fig. 2:**
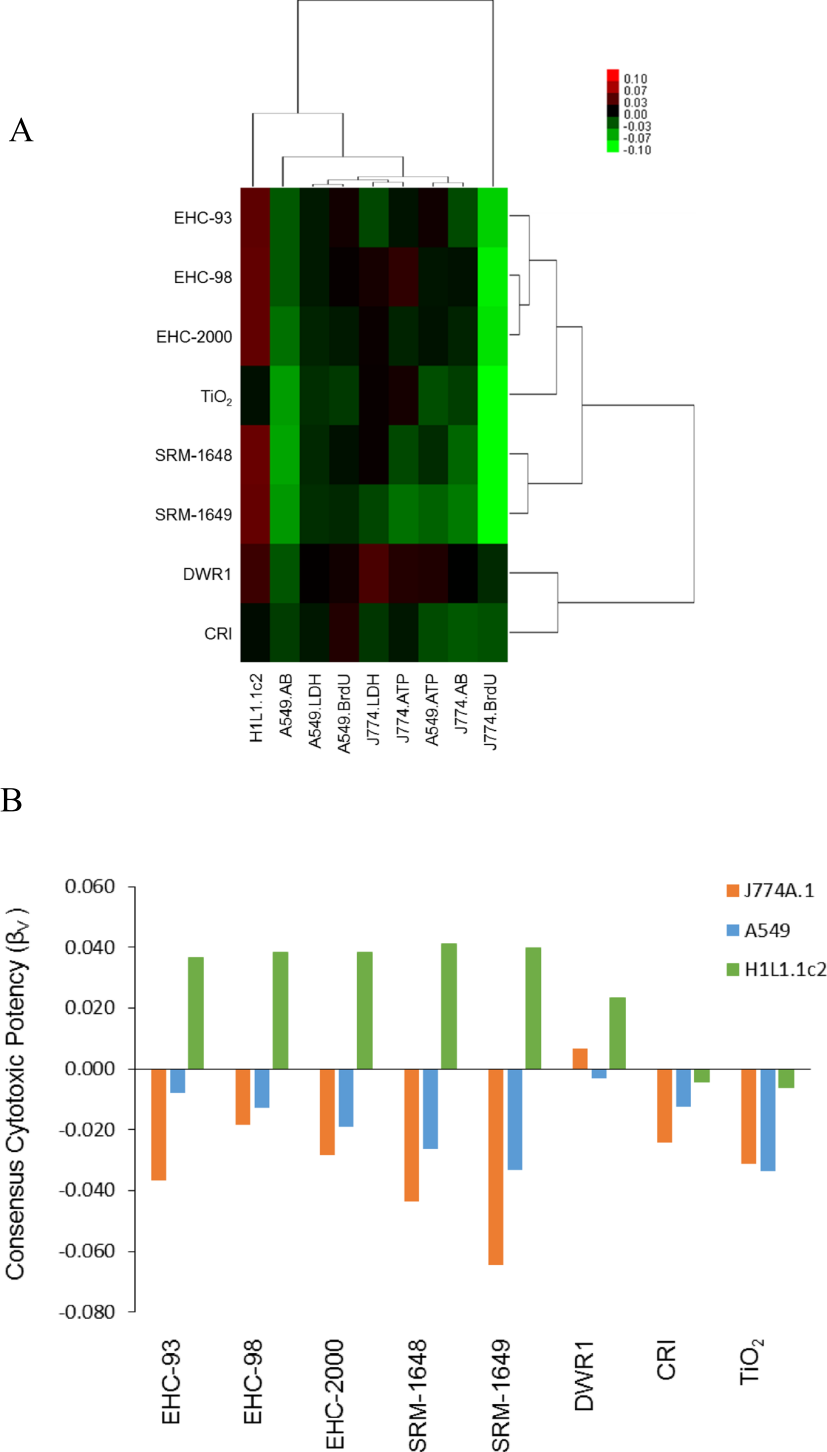
Heatmap with hierarchical cluster analysis of cytotoxic potency of particles in J774A.1 and A549 cells based on cytotoxicity assays (LDH release, AB reduction, ATP content, BrdU incorporation) and AhR induction in H1L1.1c2 cells. Within the heatmap, green color represents increased cytotoxic potency of particles (negative β) for majority of endpoints, except the AhR assay, where red color depicts increased potency (positive β) (**a**). Consensus cytotoxic potency β_V CELLS_ was calculated for each particle as average of all assay potencies, separately, for J774A.1 and A549 cells. AhR induction potencies of the particles in H1L1.1c2 cells are also presented (**b**)

Cytotoxic potency of each particle was represented by β_V_, the rate of change of dose with respect to the logarithm of fold-effect in a given assay. The potency estimates were summarized in a heatmap (Fig. 2a). In both cell lines, SRM-1648 and SRM-1649 urban particles were more cytotoxic than the other particles, including the EHC-type particles. TiO_2_ particles were also notably cytotoxic. The PM_2.5_ DWR1 and the CRI particles showed a cluster of the lowest cytotoxic potency (Fig. 2a). This behavior of CRI in the present series of experiments was atypical, as we expected CRI, based on our experimental data, to be more cytotoxic in vitro than TiO_2_. In general, there was no correlation of particle effects between the two cell lines based on the corresponding individual assays, with the exception of J774A.1 cell proliferation response which was significantly correlated with A549 cell proliferation estimate (*R* = 0.792; *p* = 0.019; Table 1).

Next, we combined cytotoxic potencies of the particles from all in vitro endpoints in a given cell line into a single β_V_ value (Fig. 2b). The cell-type specific, combined potency data outlined the cytotoxicity-based ranking of the particles and indicated that overall, J774A.1 cells were more susceptible to particle effects than A549 cells. SRM- and EHC-type urban particles had comparable potency for Ah receptor-dependent gene induction in H1L1.1c2 cells, which was markedly lower for the PM_2.5_ DWR1 particles, while a weak inhibitory potency was observed in the case of the TiO_2_ and CRI particles (Fig. 2b). Correlation analysis of the cell type-specific consensus potency β_V_ and biological reactivity of the particles β_R_ (any deviation from baseline control values) indicated that the aggregated cytotoxic responses of A549 and J774A.1 cells were significantly correlated (*p* ≤ 0.05; Table 2).

### Cytokine secretion in vitro

**Fig. 3:**
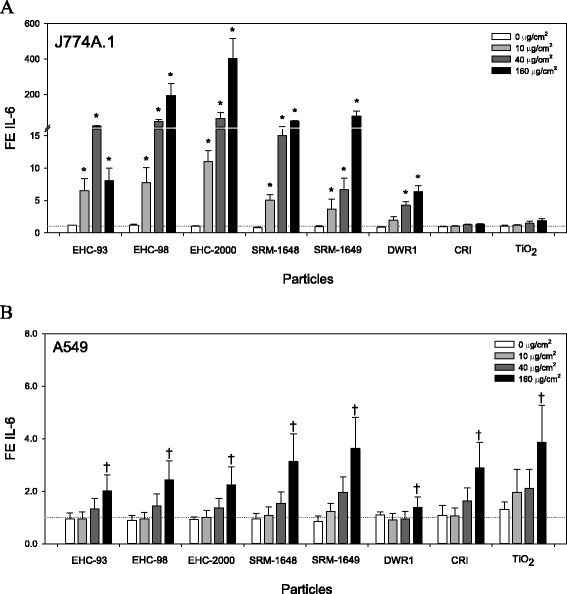
Interleukin (IL)-6 levels in cell culture supernatants from J774A.1 (**a**) and A549 (**b**) cells exposed to particles for 24 h. Values represent mean fold effect (FE) over control ± standard error of the mean (*n* = 3). Two way ANOVA; J774A.1, PM × Dose interaction, *p* < 0.001, asterisks (*) represent effects significantly different from Dose 0 control, Tukey test, *p* < 0.05; A549, Dose main effect, *p* < 0.001, Dose 160 vs. 0 or 10 (†), Tukey test, *p* < 0.05

**Fig. 4:**
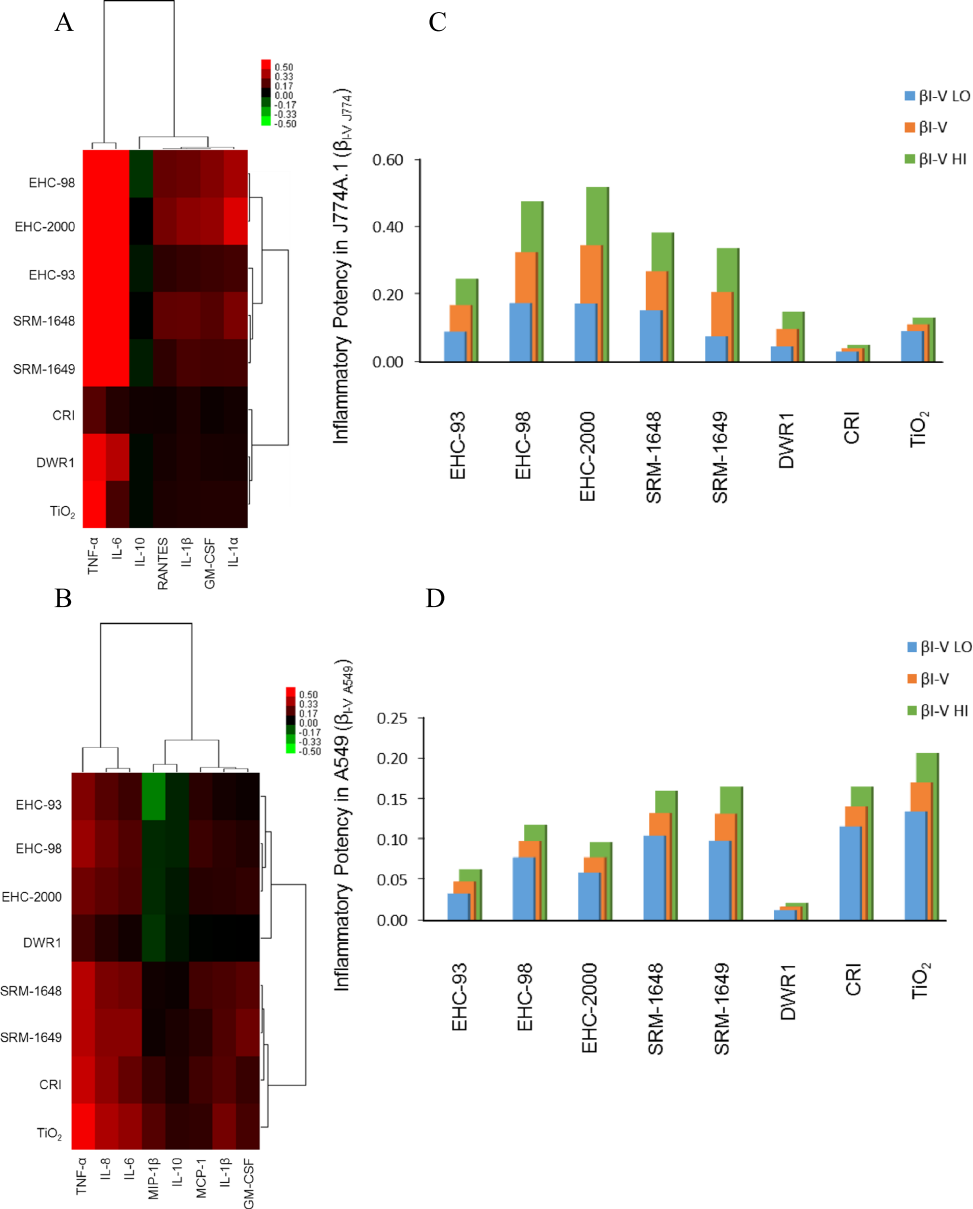
Heatmap with hierarchical cluster analysis of cytotoxic potency of particles in J774A.1 (**a**) and A549 (**b**) cells based on cytokine release after 24 h exposure to particles. Inflammatory potency estimate, β_I-V_ for J774A.1 (**c**) and A549 (**d**) cells representing the average cytokine potency adjusted for cell viability. The β_I-V LO_ represents the lower estimate (IL-10 and IL-6 potency subtracted from average potency), while β_I-V HI_ is the higher estimate (IL-10 potency subtracted, IL-6 added to average potency). Within the heatmaps, red color represents increased inflammatory potency of particles (increased cytokine/chemokine levels; positive β), whereas green color represents decreased inflammatory potency (negative β)

Release of cytokines into the culture medium upon cell exposures was taken as a measure of inflammatory potential of the particles. Significant release of cytokines was induced in response to particle exposures in both cell lines. The observed responses were particle type- and/or dose-specific as exemplified by IL-6 release (Fig. 3a and b; two way ANOVA, PM × Dose, *p* < 0.001 for J774A.1; Dose main effect, *p* < 0.001 for A549) and the other cytokines (Additional file 3: Figure S2A to S2F; Additional file 4: Figure S3A to S3G, two way ANOVA, *p* < 0.05). Cell viability-adjusted inflammatory potency (β_I-V_; cytokine release) of the particles in the cells was visualized by a heatmap. In both cell lines, the majority of the cytokines were increased in response to particle exposures, except IL-10, which generally remained unchanged or decreased in J774A.1 cells (Fig. 4a and Additional file 3: Figure S2D; two way ANOVA, PM × Dose, *p* = 0.026) and remained unchanged or showed an increasing trend in A549 cells (Fig. 4b and Additional file 4: Figure S3D; not significant). Interleukin-6 and TNF-α showed the highest fold-change from baseline values in J774A.1 cells (Fig. 3a and Additional file 3: Figure S2F two way ANOVA, PM × Dose, *p* < 0.001) and clustered separately from the rest of the cytokines (Fig. 4a). In A549 cells, IL-6, IL-8 and TNF-α showed the highest fold-change from controls (Fig. 3b, Additional file 4: Figure S3C and S3G; two way ANOVA, PM × Dose, *p* < 0.05 for IL8; PM, Dose main effects, *p* < 0.001 for TNF-α; Dose main effect, *p* < 0.001 for IL-6) and clustered separately from the remaining cytokines (Fig. 4b).

As was observed for most of the cytotoxicity endpoints, particle effects on corresponding individual cytokine release in A549 and J774A.1 cells were not correlated (Table 1).

Notably, we observed a significant correlation between AhR activity in H1L1.1c2 cells with IL-1β, IL-6 and TNF-α release in J774A.1 cells (Table 3; *R* > 0.7, *p* ≤ 0.05), but not with A549-produced cytokines.

Next, inflammatory potency β_I-V_ values for all cytokines were averaged within a cell line to obtain a consensus inflammatory index presented in three scenarios that assumed the general role of specific cytokines as anti- or pro-inflammatory mediators. The lower inflammatory estimate β_I-V LO_ was computed by subtracting potency values for IL-10 and IL-6 from the average estimate, assuming the dual role of IL-6 as a pleiotropic inflammatory mediator [31, 32], while the higher inflammatory estimate, β_I-V HI_ was obtained by subtracting IL-10 potency value only. The inflammatory index revealed a distinct profile of particle effects on cytokine release in A549 and J774A.1 cells, highlighting a difference in particle ranking.

For example, J774A.1 cells were more sensitive to the effects of urban particles, especially EHC-98, EHC-2000 (Fig. 4c) whereas A549 cells were impacted most by CRI and TiO_2_ mineral particles (Fig. 4d). Based on the inflammatory scenarios, IL-6 release was the dominant response in J774A.1 cells, with highest magnitude of effect under the β_I-V LO_ and β_I-V HI_ inflammatory scenarios, while its impact in A549 cells was minor. Correlation analysis of the consensus β_I-V_ inflammatory potency indices of the particles indicated that the aggregated inflammatory responses of the two cell lines were distinct (Table 2).

The particles were screened for the presence of endotoxin, biogenic material capable of inducing cytokines in vitro and in vivo. Majority of the urban particles, including DWR1 had similar endotoxin content (~70 – 130 EU/μg particles). SRM-1649 particles had noticeably less endotoxin (3.0 EU/μg particles) while mineral particles did not contain endotoxin (Table 4). A correlation of endotoxin with the inflammatory potency estimates in J774A.1 cells (β_I-V J774A.1_) and A549 cells (β_I-V A549_) revealed a significant correlation with the inflammatory potency estimate in J774A.1 cells (*R* = 0.729; *p* = 0.040), but not A549 cells (Table 5).

**Table 4:** Endotoxin content of particle stocks

PM	EHC-93	EHC-98	EHC-2000	SRM-1648	SRM-1649	DWR1	CRI	TiO_2_	PM buffer
Endotoxin (EU/μg PM)	100.0 ± 1.0	127.0 ± 1.0	112.0 ± 7.0	117. ± 0.4	3.0 ± 0.4	74.0 ± 10.0	ND	ND	1.0 ± 4.0

*ND* not detectable

**Table 5:** Pearson correlations for the combined in vitro and in vivo potency estimates versus endotoxin content of particles

Cytokine induction vs endotoxin	β_I-VJ774A.1_	β_I-V A549_	β_I BALB/c_
R	**0.729**	−0.557	0.020
*p* (2-tailed)	***0.040***	*0.152*	*0.963*

Consensus inflammatory potency β_I-V_ represents the average unbiased particle potency response for inducing a cytokine response inflammatory potency estimate β_I-BALB/c_ is the average of all in vivo cytokines assessed in BAL fluid

Numbers in bold represent statistically significant correlations (*p* ≤ 0.05)

### In vitro integrated particle potency

**Fig. 5:**
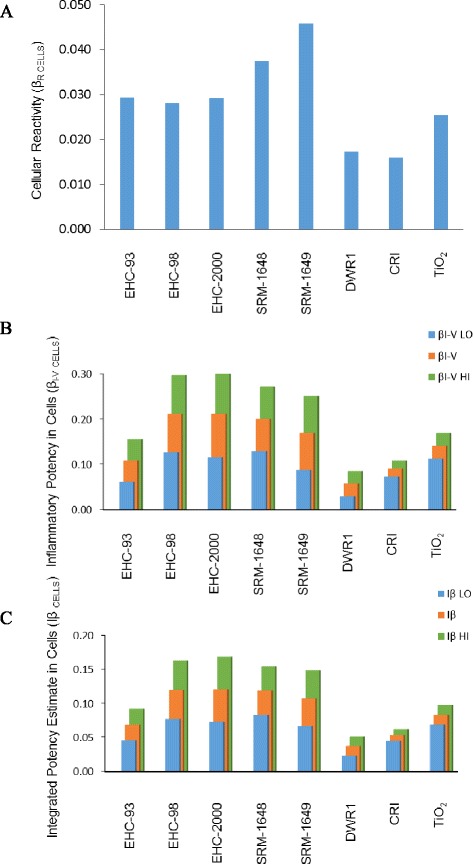
Biological reactivity estimate, β_R CELLS_ was derived by averaging the cytotoxic potencies of the particles in A549 and J774A.1 cells, as well as the AhR activity in H1L1.1c2 cells (**a**). A combined inflammatory estimate, β_I-V CELLS_, was obtained for each particle by averaging particle inflammatory potency estimates adjusted for cell viability of J774A.1 and A549 cells. The β_I-V LO_ represents the lower estimate (IL-10 and IL-6 potency subtracted from average potency), while β_I-V HI_ is the higher estimate (IL-10 potency subtracted, IL-6 added to average potency) (**b**). An integrated potency estimate of the particles, Iβ _CELLS_ was determined by averaging the biological reactivity (cytotoxic potency) and inflammatory estimates for each particle. Iβ _LO_ represents the lower estimate (IL-10 and IL-6 potency subtracted from average potency). Iβ _HI_ represents the higher estimate (IL-10 potency subtracted, IL-6 added to average potency) (**c**)

To summarize all cytotoxic particle effects in vitro across cell types, biological reactivity β_R CELLS_ was derived by averaging the absolute values of the cytotoxic potencies, β_V_ of the particles in A549 and J774A.1 cells and the AhR activity in H1L1.1c2 cells, assuming biological reactivity of the particles as any deviation from baseline. From the β_R CELLS_ estimate, SRM-1648 and SRM-1649 particles had the highest overall cell potency in contrast to DWR1 and CRI particles which showed the lowest values (Fig. 5a).

Similarly, an inflammatory potency estimate β_I-V CELLS_, was obtained for each particle by averaging the cell viability-adjusted inflammatory potency values of the particles across both cell lines for all cytokines detected, for each inflammatory scenario (Fig. 5b). The estimates impacted the magnitude of particle potency ranking, but the ranking was comparable between the different scenarios, where the urban particles, except EHC-93 were more potent than mineral particles and DWR1.

Lastly, an overall integrated potency estimate, Iβ _CELLS_ of the particles was calculated by averaging the biological reactivity, β_R CELLS_ with inflammatory indices, β_I-V CELLS_for each particle (Fig. 5c). The Iβ _CELLS_ profile was similar to the profile of the inflammatory indices β_I-V CELLS_.

### Toxicity of particles in vivo

**Fig. 6:**
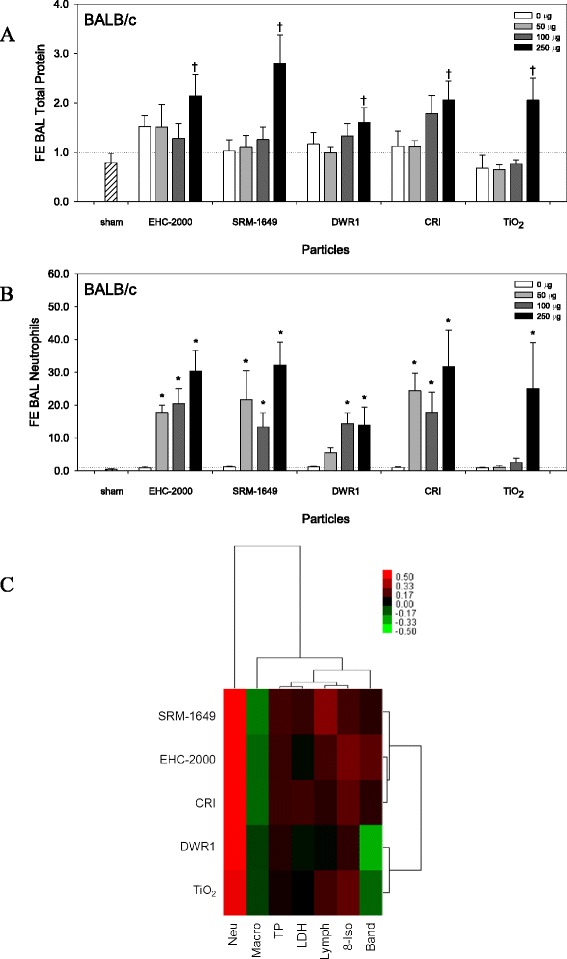
Total protein levels (**a**) in bronchioalveolar lavage (BAL) fluid of BALB/c mice exposed to particles for 24 h by intratracheal (IT) instillation. Neutrophil cell counts (**b**) were determined from cytospin preparations of BAL. All values are presented as mean fold effect (FE) over control ± standard error (*n* = 5). Two way ANOVA; Total protein, Dose main effect, *p* < 0.001, Dose 250 vs. 0, 50 or 100 (†), Tukey test, *p* < 0.001; Neutrophils, PM × Dose interaction, *p* = 0.004, asterisks (*) represent effects significantly different from Dose 0 control, Tukey test, *p* < 0.05. Heatmap with hierarchical cluster analysis of toxicity endpoints (cell differential counts and biochemical endpoints) (**c**) assessed in BALB/c mice 24 h after exposure to particles by intratracheal instillation, summarized as potency estimates (slopes of the dose response). Within the heatmaps, red color represents increased toxic potency of particles (increased biochemical measures or cell counts; positive β), whereas green color represents decreased toxic potency, with the exception of macrophages, whereby green color representing a loss of cells is a measure of increased particle potency (negative β)

BALB/c mice were exposed for 24 h to a subset of particles selected based on contrasting in vitro potency (EHC-2000, SRM-1649, CRI, TiO_2_ and DWR1) by intratracheal instillation (IT). Acute pulmonary toxicity was observed in mice exposed to the high dose (250 μg) of the particles, as shown by a general increase in BAL total protein (Fig. 6a; two way ANOVA, Dose main effect, *p* < 0.001) and BAL LDH (Additional file 5: Figure S4A; two way ANOVA, PM × Dose, *p* = 0.035). A particle dose-dependent increase in 8-isoprostane levels in blood plasma (Additional file 5: Figure S4B; two way ANOVA, Dose main effect, *p* = 0.006) indicated a systemic oxidative stress after exposure to particles. Neutrophil influx (Fig. 6b; two way ANOVA, PM × Dose, *p* = 0.004) and a drop in lung-resident alveolar macrophages (Additional file 5: Figure S4C; two way ANOVA, Dose main effect, *p* < 0.001, PM main effect, *p* = 0.018) were consistent with a particle challenge and clearance after 24 h. Based on the in vivo toxic potency estimates (β from cell differential counts and biochemical endpoints), particles elicited an increase in acute toxicity and cell injury marked by an increase in neutrophils and a decrease in macrophages. SRM-1649, EHC-2000 and CRI clustered separately from less toxic particles DWR1 and TiO_2_ (Fig. 6c).

### Cytokine secretion in vivo

**Fig. 7:**
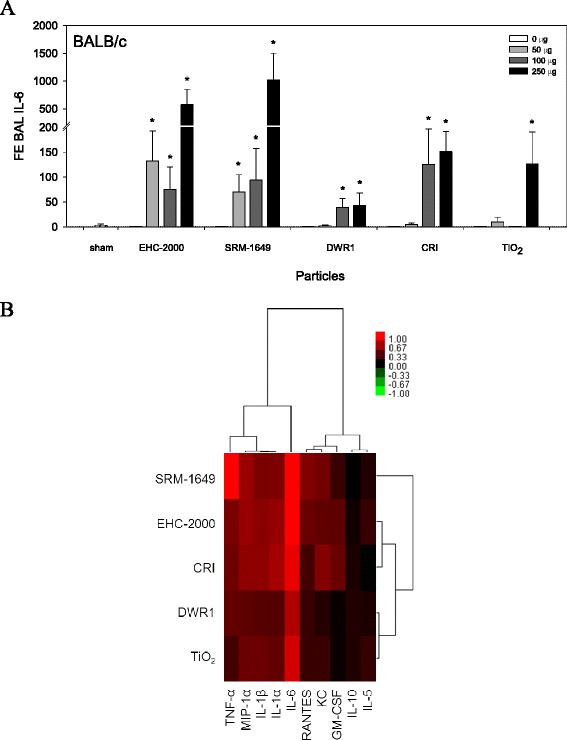
Interleukin (IL)-6 (**a**) levels in BAL. All values are presented as mean fold effect (FE) over control ± standard error (*n* = 5). Two way ANOVA; IL-6, PM × Dose interaction, *p* = 0.036, asterisks (*) represent effects significantly different from Dose 0 control, Tukey test, *p* < 0.05. Heatmap with hierarchical cluster analysis of the individual inflammatory mediators (cytokines/chemokines) (**b**) assessed in BALB/c mice 24 h after exposure to particles by intratracheal instillation, summarized as potency estimates (β from the cytokine dose response). Within the heatmaps, red color represents increased inflammatory potency of particles (increased cytokine/chemokine levels; positive β), whereas green color represents decreased inflammatory potency (negative β)

Significant particle type- and/or dose-dependent increase in inflammatory mediators IL-6, IL-1α, IL-1β, IL-5, KC, MIP-1α, RANTES and TNF-α (Fig. 7a and Additional file 6: Figure S5A to S5I); two way ANOVA, *p* < 0.05) were measured in BAL fluid collected from mice exposed to particles for 24 h, except IL-10 which was not altered (Additional file 6: Figure S5E, not significant). Individual in vivo inflammatory potency estimates (β from the cytokine dose response) confirmed a particle-induced increase for most of cytokines assessed. IL-6 and TNF-α were highlighted as the most responsive cytokines to particle exposures in vivo (Fig. 7b). SRM-1649 was the most potent particle also based on the inflammatory potency estimates, as it clustered distinctly from the remaining particles (Fig. 7b).

### In vivo integrated particle potency

**Fig. 8:**
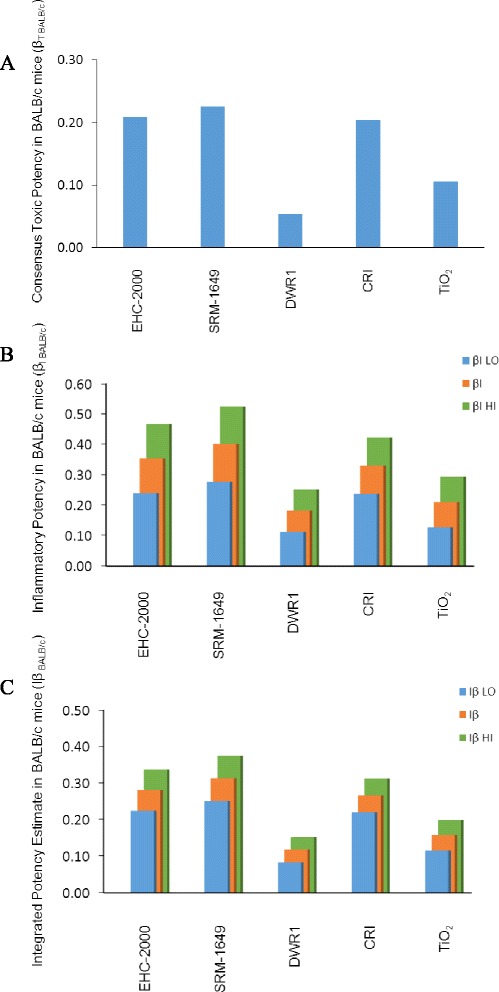
Toxic potency estimate in BALB/c, β_T__BALB/c_ was derived by averaging the potencies of the particles across the cell-specific and biochemical measures from BALB/c mice exposed to the particles by intratracheal instillation (**a**). The combined inflammatory potency estimate, β_I__BALB/c_, was obtained for each particle by averaging the particle potencies for altered cytokine/chemokine levels in bronchioalveolar lavage. The β_I LO_ represents the lower estimate (IL-10 and IL-6 potency subtracted from average potency). The β_I HI_ represents the higher estimate (IL-10 potency subtracted, IL-6 added to average potency) (**b**). The integrated potency estimate, Iβ _BALB/c_ of the particles was determined by averaging the toxic and inflammatory potency estimates for each individual particle. The Iβ _LO_ represents the lower estimate (IL-10 and IL-6 potency subtracted from average potency), while Iβ _HI_ is the higher estimate (IL-10 potency subtracted, IL-6 added to average potency) (**c**)

All of the acute toxicity endpoints assessed in vivo were averaged and depicted as β_T BALB/c_ toxicity estimate for each particle. The combined β_T BALB/c_ estimate revealed SRM-1649, EHC-2000 and CRI as highly potent particles, compared to TiO_2_ and DWR1 (Fig. 8a). An inflammatory potency estimate β_I BALB/c_ was obtained by averaging the dose response of all cytokines measured in BAL fluid. As was done for in vitro cytokines, three scenarios were described, β_I LO BALB/c_, β_I HI BALB/c_ and their average, based on subtraction of both, IL-10 and IL-6 (β_I LO BALB/c_), or IL-6 only (β_I HI BALB/c_). The inflammatory potency estimates β_I BALB/c_ ranked identically to toxicity estimates β_T BALB/c_, with no impact of the inflammatory potency scenarios on the ranking of particles (Fig. 8b). A final, in vivo integrated potency estimate Iβ _BALB/c_ was calculated by averaging β_T BALB/c_ and β_I BALB/c_ for each particle (Fig. 8c), as a consensus of all endpoints measured in vivo. Iβ _BALB/c_ estimate revealed SRM-1649, EHC-2000 and CRI as more potent than TiO_2_ and DWR1 respectively, regardless of the integrated potency estimate scenarios (Iβ _HI_, Iβ _LO_). Endotoxin content in particle preparations administered to mice was not correlated with the combined in vivo inflammatory potency estimates (β_I BALB/c_) of the particles (Table 5).

### In vitro and in vivo correlation

The relationship between particle effects in cells in vitro and in BALB/c mice exposed to particles by intratracheal instillation was explored by correlating the individual in vitro and in vivo particle potency estimates, as well as the in vitro and in vivo combined particle potency estimates for the five particles tested by both approaches.

Majority of the individual particle potency estimates for the corresponding endpoints were not correlated, with the exception of a single association; the cellular LDH content in J774A.1 cells, significantly correlated with LDH content measured in BALB/c BAL fluid, (Additional file 1: Table S2; *R* = 0.928, *p* = 0.023).

**Fig. 9:**
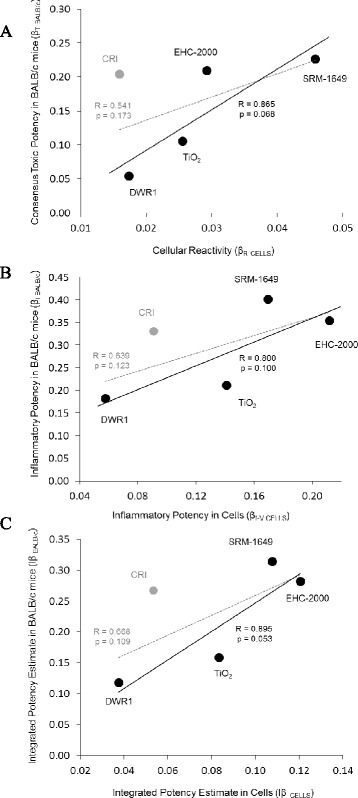
Scatter plots of key corresponding in vitro and in vivo combined particle potency estimates β_R CELLS_ (in vitro assays) with β_T BALB/c_ (in vivo cell counts and biochemical endpoints) (**a**); β_I-V CELLS_ (in vitro cytokines) with β_I BALB/c_ (in vivo cytokines) (**b**) and integrated particle potency estimates, Iβ _CELLS_ (averaged in vitro assays and cytokines) with Iβ _BALB/c_(average of in vivo cell counts, biochemical endpoints and cytokines) (**c**). Pearson correlation coefficients and p-values (1-tailed) are presented for correlations of all particles (*dashed grey line*) and particles excluding CRI (*solid black line*)

When the combined particle potency estimates in vitro and in vivo were assessed by Pearson product–moment correlation analysis for all particles tested, no statistically significant correlations were observed between the corresponding pairs of the key estimates, including the key estimates; β_R CELLS_ versus β_T BALB/c_ (Fig. 9a and Additional file 1: Table S3; *R* = 0.541, *p* = 0.173); β_I-V CELLS_ versus β_I BALB/c_ (Fig. 9b and Additional file 1: Table S3; *R* = 0.639, *p* = 0.123) and Iβ _CELLS_ versus Iβ _BALB/c_ (Fig. 9c, Additional file 1: Table S3; *R* = 0.668, *p* = 0.109). However, a consistent increasing trend between these in vitro and in vivo estimates was observed across the particles, where DWR1 was ranked low, CRI and TiO_2_ were ranked intermediate and SRM-1649 and EHC-2000 were ranked high in potency (Fig. 9). Based on the observed trend in the ranking of the particles, a directional (one-tailed) Spearman’s rank-order correlation analysis was conducted. The analysis revealed that while the combined potency estimates β_R CELLS_ versus β_T BALB/c_ remained non-correlated (Table 6; *R* = 0.667, *p* = 0.109), correlations of β_I-V CELLS_ versus β_I BALB/c_ and the integrated potency estimates Iβ _CELLS_ versus Iβ _BALB/c_ approached the threshold for significance α = 0.05 (Table 6; *R* = 0.8, *p* = 0.052). Finally, improved correlation coefficients for the associations of the combined particle potency estimates were observed when mineral particle responses were excluded from the estimates, albeit they were not statistically significant due to low power (Additional file 1: Table S3); β_R CELLS_ versus β_T BALB/c_ (*R* = 0.862, *p* = 0.169); β_I-V CELLS_ versus β_I BALB/c_ (*R* = 0.888, *p* = 0.153) and Iβ _CELLS_ versus Iβ _BALB/c_ (*R* = 0.955, *p* = 0.096). Remarkably, CRI contributed most to the decreased association of in vitro and in vivo potency estimates, as exclusion of CRI only revealed improved correlation comparable to the associations excluding both mineral particles (Fig. 9, Additional file 1: Table S3); β_R CELLS_versus β_T BALB/c_ (*R* = 0.865, *p* = 0.068); β_I-V CELLS_ versus β_I BALB/c_ (*R* = 0.800, *p* = 0.100) and Iβ _CELLS_ versus Iβ _BALB/c_ (*R* = 0.895, *p* = 0.053). When the Spearman’s rank-order correlation analysis was conducted on the particle ranks while excluding CRI, a statistically significant rank-order was observed for β_R CELLS_ versus β_T BALB/c_(Table 6; *R* = 1.0, *p* < 0.001), while β_I-V CELLS_ versus β_I BALB/c_and the integrated potency estimates Iβ _CELLS_ versus Iβ _BALB/c_ did not show a significant trend in a correlation due to low power (Table 6; *R* = 0.8, *p* = 0.100).

**Table 6:** Rank order correlations for the combined in vitro (cell lines) and in vivo (BALB/c mouse) particle potency estimates for all particles (PM) and excluding cristobalite (CRI)

Particle potency estimates	Particles	Ranking in vitro		Ranking in vivo		Rank-order correl. (All PM)^a^		Rank-order correl. (no CRI)^b^	
						R	p (1-tailed)	R	p (1-tailed)
Toxicity β_R CELLS_ vs. β_T BALB/c_	CRI	5	-	3	-				
	DWR1	4	(4)	5	(4)				
	EHC-2000	2	(2)	2	(2)	0.667	*0.109*	**1.000**	***<0.001***
	SRM-1649	1	(1)	1	(1)				
	TiO_2_	3	(3)	4	(3)				
Inflammation β_I-V CELLS_ vs. β_I BALB/c_	CRI	4	-	3	-				
	DWR1	5	(4)	5	(4)				
	EHC-2000	1	(1)	2	(2)	0.800	*0.052*	0.800	*0.100*
	SRM-1649	2	(2)	1	(1)				
	TiO_2_	3	(3)	4	(3)				
Integrated Potency Iβ _CELLS_ vs. Iβ _BALB/c_	CRI	4	-	3	-				
	DWR1	5	(4)	5	(4)				
	EHC-2000	1	(1)	2	(2)	0.800	*0.052*	0.800	*0.100*
	SRM-1649	2	(2)	1	(1)				
	TiO_2_	3	(3)	4	(3)				

Particle ranking based on integrated particle potency estimates across cell types and in vitro or in vivo endpoints, where the lowest number represents the most potent particle

The ranking outside brackets includes all PM, the ranking within brackets excludes CRI

^a^Spearman’s rank-order correlation and *p*-values (1-tailed) on ranked corresponding in vitro and in vivo potency estimates for all PM, ^b^excluding CRI

Numbers in bold represent statistically significant correlations (*p* ≤ 0.05)

## Discussion

In this work we examined the level of concordance between in vitro and in vivo responses to particulate matter, to determine the potential of cell-based cytotoxicity assays and cytokine/chemokine mediators in screening the toxicity of ambient particles. The dose response results from the bioassays conducted in cell lines and from intratracheal exposures of mice to particles were summarized as slope-based measures of particle potency for each endpoint. The potency values were combined within each exposure model to obtain model-specific consensus potency estimates of toxicity and inflammatory potential of the particles, which were further combined into an overall integrated potency estimate. The key derived particle potency estimates were then assessed for correlation across the in vitro and in vivo models.

We note that in the present series of experiments we observed an atypically low in vitro cytotoxicity of the CRI sample by comparison to TiO_2_. In vivo toxicity of the same sample however was high, in accordance with our historical observations and literature data on toxicity of silica. Identity of the CRI samples was reconfirmed by ICP-MS/AES analysis of the archived, diluted working particle stocks that were used in the experiments. We have also carefully validated the datasets, but could not explain this atypical behavior of CRI in the series of in vitro assays presented here, e.g. from computational errors or material switching in assays, and therefore we report the data as observed.

Urban and mineral particles induced toxicity in vitro and in vivo (Figs. 2a and 6c). Cytotoxic responses in vitro based on the individual endpoints were in general different between epithelial cells and macrophages, with the exception of the cell proliferation response (Table 1). The pulmonary alveolar epithelium (represented by the A549 cell line) serves as a barrier/interface with the outside environment, facilitates gas transfer and fluid clearance, reduces surface tension and prevents exposure to particles, toxicants and microbes [33]. It is a resilient interface adapted to the mechanical forces of ventilation and it is involved in pulmonary innate host defense and tissue repair [34]. Alveolar epithelial cells also produce a multitude of factors such as cytokines and chemokines (e.g. MCP-1, GM-CSF, IL-8), which impact the activity of pulmonary macrophages [35]. As well, the cells express a variety of pattern recognition receptors (e.g. NLRs, TLRs) for microbes and particulates [34]. Pulmonary macrophages are phagocytic cells involved in direct response to pathogens, antigen sensing and activation of acquired and innate immunity, and they serve as modulators of inflammation through removal of pathogens, foreign materials and apoptotic cells, and through production of a specific suite of pro- and anti-inflammatory cytokines and chemokines [34]. We used the J774A.1 murine macrophage cell line as a model macrophage. Although the cells originate from peripheral blood, they display the hallmarks common to both blood-borne and lung-resident macrophages, including adherent, cytologic and phagocytic properties [36].

The greater susceptibility of the macrophages to particle exposures compared to the epithelial cells observed in this work (Fig. 3a) highlights the importance of using functionally distinct cell types for hazard screening. The innate phagocytic activity of macrophages results in increased intracellular particle loads and cytotoxic effects that are more pronounced, than in epithelial cells which do not undergo internalization of particles to the same extent. Specifically, J774A.1 cell proliferation was more impacted by particle exposures than A549 cells (Additional file 2: Figure S1E and S1F). A greater decrease in proliferation and increase in apoptosis in J774A.1 cells compared to A549 cells has also been demonstrated after exposures to Mexico City PM_10_particles [37]. Similarly, we have observed greater sensitivity of J774A.1 cells than A549 cells to carbon nanotube exposures [38].

The impact of increased sensitivity of J774A.1 cells to particle exposures was also revealed by a small decrease of LDH release below control levels in cells exposed to the intermediate doses of the urban particles (Fig. 1a). A combination of the decreased cell proliferation from the effects of the intermediate particle doses and a lack of space for cell division in the nearly confluent wells throughout the exposures likely resulted in an increased cell death. The phenomenon was not observed in the unexposed controls, or in cells exposed to the high doses of the particles where the overt cytotoxicity led to high LDH release, or in A549 cells that were less sensitive to particle exposures.

Nevertheless, when the panel of in vitro toxicity endpoints (viability assays) was combined, the overall particle potency was correlated across the cell lines (Table 2) regardless of their functional difference, suggesting that the cytotoxic profile of the particles reflects a more general outcome (e.g. apoptosis), over different but converging mechanisms of cytotoxicity. Although the particles affect the cells through different pathways, with a sufficiently broad array of assays it appears that the inherent cytotoxic potential of the particles can be captured. This is consistent with our previous observations that cytotoxicity in vitro appears to correlate with time-weighted average exposure limits for particles of occupational health relevance [39].

In the present study, however, inflammatory potential determined from the release of cytokines did not correlate between the two cell lines, or with the overall cytotoxic potency of the particles (Table 2). This reveals that functional impacts of particles on the cells (e.g. inflammatory response) are distinct from overt cytotoxicity and will be sensitive to the cellular model used. Similarly, we have shown previously that the impacts of urban particles and minerals on the respiratory burst function of stimulant-induced primary rat macrophages were not correlated with their cytotoxic effect determined by the water-soluble tetrazolium salt XTT reduction assay [39]. In the present study, the cytokine profiles were more reflective of the functional specificity of the cell types. In this context, A549 epithelial cells were similarly responsive to mineral and urban air particles, in contrast to J774A.1 macrophages which were more impacted by the compositionally-complex urban air particles (Fig. 4). The higher cytotoxicity of minerals in A549 cells compared to J774A.1 cells highlights a potential importance of particle surface interactions with cells that provide a barrier-type function, such as lung epithelial cells. Irregularity of insoluble particle surface at the molecular level can modulate particle cytotoxicity via surface-associated free-radical formation [40]. Assessment of inflammatory mediators was more reflective of organic content of particles. Of note is that the AhR activity in H1L1.1c2 cells was correlated with IL-1β, IL-6 and TNF-α release in J774A.1 cells (Table 3). As shown here, and previously [24], AhR agonists, such as polycyclic aromatic hydrocarbons (PAHs) are a significant component of particulate emissions. Besides its role in xenobiotic metabolism, the Ah receptor is involved in immunity and lung inflammation [41].

It has been shown that 2,3,7,8-tetrachlorodibenzo-p-dioxin (TCDD)-activated AhR signaling participates in the up-regulation of IL-1β, IL-6 and IL-8 expression through activation of the nuclear factor (NF-kB) and extra-cellular stimulus-activated kinase (ERK) signaling cascades. Dioxins and PAHs are major components of cigarette smoke. The AhR protein expression was up-regulated in the presence of TNF-α and smoking and exposure to TCDD enhanced rheumatoid arthritis (RA) inflammatory processes in synovial tissue from RA patients [42]. Interestingly, SiO_2_-induced acute lung inflammation is more severe, while fibrotic response is attenuated in AhR^−/−^ mice compared with C57Bl/6 mice, suggesting a complex role for AhR in regulation of inflammation [43]. The correlation with key pro-inflammatory cytokines produced by J774A.1 cells upon particle exposures in the present study (Table 3) suggests that AhR signaling in lungs may represent an important pathway in regulating inflammation triggered by emission particles. This notion requires further mechanistic testing. Local production of inflammatory mediators is a hallmark of pulmonary response to foreign materials. These observations emphasize the need to capture a diverse set of endpoints that are broadly reflective of the functional specificity of cells and are tied to the particular hazard or route of exposure.

We have observed a small decrease in AhR-mediated gene activity below control levels in cells exposed to CRI and TiO_2_ particles (Fig. 1c), suggesting an inhibitory effect of the particles in the Ah-dependent gene induction assay. Clarification of supernatants or cell lysates is a recommended approach to facilitate fluorimetric-, colorimetric-, or luminescence-based in vitro toxicological assessments of optically/chemically active materials such as nanoparticles, or their micron-sized counterparts [28]. This assay protocol was adapted by introducing a step to clarify cell lysates from particle residues by centrifugation to prevent potential interference with the luciferase-mediated reaction. Nonetheless, trace amounts of particles may have remained in the cell lysates allowing for the possibility of a small underestimation of AhR-mediated gene activity. Furthermore, a recent publication based on an *in silico* model for Ah receptor docking by ligands provides some evidence that TiO_2_-based nanoparticles could directly bind/adsorb to the Ah receptor with very high affinity, although this notion has yet to be experimentally validated in cellular models [44].

Endotoxin levels in urban particles were correlated with the inflammatory index β_I-V J774A.1_ which is based on the cytokine release by J774A.1 cells, but not with A549-cell based inflammatory index β_I-V A549_ or the in vivo inflammatory index β_I BALB/c_ (Table 5). It is conceivable that the endotoxin component in urban particles may play a role in cytokine induction by macrophages. Macrophages express lipopolysaccharide-responsive Toll-like receptor 4 on their cell surface [45]. Endotoxin-associated induction of pro-inflammatory cytokines TNF-α, IL-1 and IL-6 can be inhibited by the antimicrobial agent polymyxin B in alveolar macrophages exposed to urban particles [46]. However, only a small amount of endotoxin (~0.1 EU/mg particle) was detected in the urban particles tested (Table 4). The trace levels in EHC-93 particles (a particle used in the present study) do not have appreciable effects on mRNA expression of pro-inflammatory genes in human bronchial epithelial cells [47]. Also, endotoxin at comparable levels did not activate the bone marrow (measure of systemic inflammatory response) of rabbits upon its instillation in lungs of the animals [48]. In addition, in the present study, mineral particles were highly potent in triggering cytokine release both in vitro and in vivo despite the absence of endotoxin (Figs. 4 and 7). Conversely, SRM-1649 particles had ~35-fold lower endotoxin content than the other urban particles, but SRM-1649 induced cytokines at the same magnitude as in cells exposed to the other urban particles. Thus other particle components likely contributed to the release of inflammatory mediators by cells observed in our study. Up-regulation of genes for cytokines and chemokines in cells in vitro (e.g. bronchial epithelial cells) has been observed in response to various subcomponents of particulate matter including carbon-core, transition metals or polyaromatic hydrocarbons [49].

With the exception of the mineral particles, the coarse and fine urban particles tested here represent complex mixtures containing both soluble and insoluble fractions, which will contribute to the potency in vitro to varied extents due to differences in uptake mechanisms, molecular targets of the various particle components, corona formation or differences in the settling rate of the denser particle components. Therefore, homogeneity of particle suspension delivered to the cells was achieved by sonication of the particles prior to delivery. However, it has to be noted that, even with the in vivo delivery of environmental particles, similar differences of soluble and insoluble fractions of the particulate mixture will be encountered. Specifically, the issue of dilution of soluble components of ambient particles in the culture medium has been examined in previous work [24]. By ensuring internal consistency in assay conditions, the biases, if present, will be systematic, affecting all particles studied.

Instillation of particles in lungs of BALB/c mice led to elevated neutrophil counts and cytokine levels in BAL fluid, and generally unremarkable changes in biochemical markers, a profile consistent with mild, acute pulmonary inflammation at 24 h post-exposure. This was accompanied by a decrease in alveolar macrophages in BAL fluid, consistent with macrophage-associated pulmonary clearance of foreign particles (Fig. 6). Determination of neutrophil counts in lung lavage fluid represents a key, sensitive response to pulmonary instillation of urban and mineral particles [50, 51]. Recruitment of neutrophils in response to acute particle exposure is a consequence of a local lung inflammatory cascade aimed at the clearance of the foreign materials and macrophage apoptotic bodies. In our study, production of the inflammatory mediators accompanied the observed cellular differential profile measured in BAL fluid upon instillation with particles (Fig. 7). The in vivo response to particles was dominated by IL-6 and TNF-α release, along with other cytokines, including KC (murine IL-8 homolog), indicators of the presence of acute inflammation. Increases in BAL TNF-α, IL-6 and KC have been also observed by others in C57Bl/6 J mice instilled with fine and coarse urban particles collected from European cities [52].

A European study conducted using particles collected in different seasons from urban centers along with EHC-93 Ottawa urban particles examined the role of physicochemical particle properties in modulating the production of cytokines in vitro and on in vivo inflammation in rat lungs as well as adjuvant potency in allergic mouse models. The in vitro assays of cytokine production (IL-6, IL-8, TNF-α, MIP-2) in macrophage and lung epithelial cells exposed to coarse and fine particles for 20 h correlated with in vivo inflammation endpoints (TNF-α, MIP-2, albumin, Clara cell protein 16) measured in BAL of rats at 24 h after intratracheal exposure to the same particles. In contrast, poor correlation was observed between different endpoints from in vivo allergy models and inflammatory parameters, suggesting that a variety of endpoints are needed to assess the potential hazard of particles [19].

In the present study, we have observed a significant correlation of LDH content in J774A.1 macrophages with LDH in BALB/c lung lavage (Additional file 1: Table S2). Such relationship can be conceptually simpler to understand, i.e. increased potential of particles to perturb macrophage cell membranes in vitro correlates with increased potential to damage cell membranes of particle-laden lung macrophages and neutrophils, however further study is needed to ascertain the mechanisms. Therefore, we have combined the in vitro endpoints in a manner to capture the general cytotoxicity of the particles and the functionally-specific aspects (inflammatory potential) in a meaningful manner that can be subsequently deconstructed to explore the mechanisms of action of the variety of particles, and to facilitate the comparison to in vivo responses.

Our combined estimates of particle potency in vitro and in vivo for toxicity; (β_R_, β_T_) were not significantly correlated. However, the combined potency estimates for inflammatory potential (β_I-V,_ β _I_) and the integrated particle potency estimates (Iβ) that further combined the toxicity and inflammatory bioassay response potency estimates approached the threshold for significance α = 0.05 in a correlation (Table 6; *R* = 0.8, *p* = 0.052). The lack of strong associations may be due to insufficient power to obtain statistical significance. Although eight particles (with six urban particles) were screened in vitro, for workload reasons a subset of five particles only (including three urban particles) was tested in vivo, an issue that highlights the impracticality of in vivo assays for high-throughput testing. A better correlation may also have been achieved with additional in vivo time points, capturing different stages of the transient particle-induced in vivo toxicity and inflammation profile. The use of a single species could also have potentially led to higher in vitro/in vivo correlations. However, employing different, routinely used species (mouse and human) enabled the assessment of whether integrating toxicity endpoints originating from widely varied cell types (functionality and species differences) would constitute a robust in vitro surrogate that can be useful in predicting in vivo responses of biological and health relevance. As previously seen, the ranking of potencies of a series of particles was comparable between the mouse macrophage cell line J774A.1 and the human cell line THP-1 (unpublished observations). There are other variables beyond species that affect potency estimates, such as cell seeding or monolayer density at time of exposure, etc.

As illustrated by our results in vitro, toxicological responses of cells to particles of complex composition, such as urban particles may be governed by mechanisms distinct from responses to particles that are more homogeneous, such as minerals. The array of potential mechanisms that may reflect the responses of vastly different particle types may not have been adequately captured in our panel of assays. Nevertheless, the more potent materials that ranked highly in vitro were also highly ranked in vivo, while the less potent particles were ranked low, except TiO_2_ and CRI particles that were less consistent in vitro and in vivo (Fig. 9). Retesting the model with more particles of comparable physicochemical complexity (i.e. urban particles) and additional relevant assays may provide an improved model performance.

## Conclusion

The challenge with investigating the relative risk to health of different particulate source emissions is the reliability of the cytotoxicity models applied, with respect to sensitivity of the cells and potential bias for specific potency determinants. A test system should be sensitive to most if not all potency determinants in particles of different origins and should be relevant to the pathways of toxicity in humans leading to adverse health outcomes. In order to achieve this, computational approaches are needed, to reduce the complex datasets, dose responses, multiple assays and cell lines into meaningful descriptors of potency. Therefore, an approach which combines tissue-specific, functionally distinct cell types with a diverse set of assays that capture convergent toxicity pathways and risk-specific mechanisms forms an ideal basis in a tiered strategy for hazard identification and toxicity testing. It remains difficult to broadly capture most of the key events that underlie adverse pulmonary reactions. This may require the inclusion of a greater variety of assays, such as air-liquid interface models, more cell types and particles, or more relevant particles in the model.

More physiologically relevant cell-cell interaction-based models (e.g. co-cultures, or 3D spheroids grown on reconstituted basement membranes) show promise in capturing additional factors that may narrow the gap between in vitro and in vivo testing [53, 54], the latter representing the currently accepted basis of risk assessment regimes. By design, in vitro-based systems are highly relevant for high-throughput applications (e.g. EPA ToxCast, Tox21 initiatives) and high-content data (genomic, proteomic profiling) which are required to address the current challenge posed by the large number of data-poor chemicals, that must undergo toxicological risk-based prioritization and assessment. More comprehensive evaluations of the predictive potential of in vitro assays for disease-relevant adverse outcomes will be needed for in vitro assays to become a trusted and practical tool in regulatory risk assessment. Although in vitro screening approaches may not yet fully replace the need for in vivo regulatory toxicology testing, they could provide an effective component of a multi-tiered testing approach.

## Abbreviations

AhR, aryl hydrocarbon receptor; AMR, ATP-monitoring reagent; ANOVA, analysis of variance; ATCC, American type culture collection; ATP, adenosine triphosphate; BRDU, 5-bromo-2'-deoxyuridine; BSA, bovine serum albumin; CRI, cristobalite; DMEM, Dulbecco’s modified Eagle’s medium; DWR1, Downsview reference 1; EHC, Environmental Health Centre; EIA, enzyme immunoassay; EPA, Environmental Protection Agency; ERK, extra-cellular stimulus-activated kinase; EU, endotoxin units; FBS, fetal bovine serum; FE, fold effect; FGF, fibroblast growth factor; G-CSF, granulocyte colony-stimulating factor; GM-CSF, granulocyte macrophage colony-stimulating factor; HEPA, high-efficiency particulate arrestance; ICP-MS/AES, inductively coupled plasma-mass spectrometry/ atomic emission spectroscopy; IFN, interferon; IP-10, IFN-γ Inducible Protein 10; KC, keratinocyte chemoattractant; LDH, lactate dehydrogenase; M199, medium 199; MCP-1, monocyte/macrophage chemoattractant protein; MEM, minimum essential medium Eagle; MIP-1, macrophage inflammatory protein; NF-kB, nuclear factor kB; NIST, National institute of standards and technology; NLR, nucleotide oligomerization domain-like receptor; PBS, phosphate-buffered saline; PDGF, platelet-derived growth factor; PM, particulate matter; RA, rheumatoid arthritis; RANTES, regulated on activation, normal T cell expressed and secreted; SiNPs, silicon dioxide nanoparticles; SRMs, standard reference materials; TCDD, 2,3,7,8-Tetrachlorodibenzodioxin; TLR, Toll-like receptor; TMB, 3,3',5,5'-tetramethylbenzidine; TNF-α, tumor necrosis factor alpha; TSP, total suspended particulates; VEGF, vascular endothelial growth factor; XTT, 2,3-bis-(2-methoxy-4-nitro-5-sulfophenyl)-2H-tetrazolium-5-carboxanilide

## Additional files

Additional file 1: Table S1. Elemental composition and size of the urban particulate matter and mineral particles. **Table S2.** Pearson correlations for cytotoxic potency and cytokine inductions in cell lines versus BALB/c mice exposed to particles. **Table S3.** Pearson correlations for the combined average in vitro (cell lines) and in vivo (BALB/c mice) particle potency estimates. (DOC 77 kb)

Additional file 2: Figure S1. Resazurin reduction by J774A.1 (A) and A549 (B) cells exposed to particles. Average fold-effect (FE) over control ± standard error values are shown (*n* = 5). ATP content of J774A.1 (C) and A549 (D) cells exposed to particles. (*n* = 3). Proliferation in J774A.1 (E) and A549 (F) cells exposed to particles. (*n* = 3). Two way ANOVA; Resazurin assay, J774A.1, PM main effect, *p* < 0.001, SRM-1648 vs. DWR1, EHC-98 or EHC-2000 (†), SRM-1649 vs. DWR1, EHC-93, EHC-98, EHC-2000, TiO_2_ or CRI (‡), Tukey test, *p* < 0.05, Dose main effect, *p* < 0.001, Doses 0, 10 or 20 vs. 40, 80 or 160 (#), Dose 40 vs. 80 or 160 (not shown), Dose 80 vs. 160 (not shown), Tukey test, *p* < 0.05; Resazurin assay, A549, PM × Dose interaction, *p* = 0.001, asterisks (*) represent effects significantly different from control, Tukey test, *p* < 0.05. ATP assay, J774A.1, PM main effect, *p* < 0.001, EHC-98 vs. SRM-1648, SRM-1649 or EHC-2000 (†), SRM-1649 vs. DWR1, TiO_2_ or EHC-93 (‡), Tukey test, *p* < 0.05, Dose main effect, *p* < 0.001, Dose 160 vs. all doses (#), Tukey test, *p* < 0.001; ATP assay, A549, PM main effect, *p* < 0.001, DWR1 vs. all particles (†), EHC-93 vs. SRM-1649, CRI or TiO_2_ (‡), Tukey test, *p* < 0.05, Dose main effect, *p* < 0.001, Dose 10 vs. 0, 40, 80 or 160 (not shown), Dose 20 vs. 80 (not shown), Dose 160 vs. 0, 20, 40 or 80 (#), Tukey test, *p* < 0.05. BrdU assay, J774A.1, PM main effect, *p* < 0.001, DWR1 or CRI vs. EHC-93, EHC-98, EHC-2000, SRM-1648, SRM-1649 or TiO_2_ (†), Tukey test, *p* < 0.05, Dose main effect, *p* < 0.001, Doses 0 or 10 vs. 20, 40, 80 or 160 (‡), Doses 20 or 40 vs. 80 or 160 (not shown), Dose 80 vs. 160 (not shown), Tukey test, *p* < 0.05; BrdU assay, A549, Dose main effect, *p* < 0.001, Dose 10 vs. 0, 20, 80 or 160 (†), Dose 40 vs. 80 or 160 (‡), Tukey test, *p* < 0.05. (DOCX 80 kb)

Additional file 3: Figure S2. Granulocyte-Macrophage Colony-Stimulating Factor (GM-CSF) (A), interleukin (IL)-1α (B), IL-β (C), IL-10 (D), Regulated upon Activation Normal T cell Expressed and Secreted (RANTES) (E) and Tumor Necrosis Factor (TNF)-α (F) levels in cell culture supernatants of J774A.1 cells exposed to particles for 24 h. Values are presented as mean fold-effect (FE) ± standard error (*n* = 3). Two way ANOVA; GM-CSF, PM × Dose interaction, *p* = 0.001, asterisks (*) represent effects significantly different from Dose 0 control, Tukey test, *p* < 0.05; IL-1α, PM × Dose interaction, *p* < 0.001, asterisks (*) represent effects significantly different from Dose 0 control, Tukey test, *p* < 0.05; IL-β, PM × Dose interaction, *p* < 0.001, asterisks (*) represent effects significantly different from Dose 0 control, Tukey test, *p* < 0.05; IL-10, PM × Dose interaction, *p* = 0.026, asterisks (*) represent effects significantly different from Dose 0 control, Tukey test, *p* < 0.05; RANTES, PM × Dose interaction, *p* < 0.001, asterisks (*) represent effects significantly different from Dose 0 control, Tukey test, *p* < 0.05; TNF-α, PM × Dose interaction, *p* < 0.001, asterisks (*) represent effects significantly different from Dose 0 control, Tukey test, *p* < 0.05. (DOCX 73 kb)

Additional file 4: Figure S3. Granulocyte-Macrophage Colony-Stimulating Factor (GM-CSF) (A), interleukin (IL)-1β (B), IL-8 (C), IL)-10 (D), Monocyte Chemoattractant Protein (MCP)-1 (E), Macrophage Inflammatory Protein (MIP)-1β (F) and Tumor Necrosis Factor (TNF)-α (G) levels in cell culture supernatants of A549 cells exposed to particles for 24 h. Values are presented as mean fold-effect (FE) ± standard error (*n* = 3). Two way ANOVA; GM-CSF, Dose main effect, *p* < 0.001, Dose 160 vs. 0, 10 or 40 (†), Dose 10 vs. 40 (not shown), Tukey test, *p* < 0.05; IL-1β, PM main effect, *p* = 0.004, TiO_2_ vs. DWR1 or EHC-93 (†), Tukey test, *p* < 0.05, Dose main effect, *p* < 0.001, Doses 40 or 160 vs. 0 or 10 (‡), Tukey test, *p* < 0.05; IL-8, PM × Dose interaction, *p* = 0.016, asterisks (*) represent effects significantly different from Dose 0 control, Tukey test, *p* < 0.05; IL-10, not statistically significant; MCP-1, PM × Dose interaction, *p* = 0.001, asterisks (*) represent effects significantly different from Dose 0 control, Tukey test, *p* < 0.05; MIP-1β, PM main effect, *p* = 0.014, TiO_2_ vs. EHC-93 (†), Tukey test, *p* < 0.05, Dose main effect, *p* = 0.023, Dose 160 vs. 10 (‡), Tukey test, *p* < 0.05; TNF-α, PM main effect, *p* < 0.001, TiO_2_ vs. DWR1, EHC-93 or EHC-2000 (†), CRI vs. DWR1 (‡), Tukey test, *p* < 0.05, Dose main effect, *p* < 0.001, Dose 0 vs. 10, 40 or 160 (#), Dose 10 vs. 40 (not shown), Dose 160 vs. 10 or 40 (not shown), Tukey test, *p* < 0.05. (DOCX 85 kb)

Additional file 5: Figure S4. Lactate dehydrogenase (LDH) levels in bronchioalveolar lavage (BAL) fluid (A), 8-isoprostane levels in blood plasma (B), Macrophage (C), Band cell (D) and lymphocyte (E) counts in BAL fluid from BALB/c mice exposed to particles by intratracheal instillation, at 24 h post-exposure. Values represent mean fold-effect (FE) ± standard error of the mean (*n* = 5). Two way ANOVA; LDH, PM × Dose interaction, *p* = 0.035, asterisks (*) represent effects significantly different from Dose 0 control, Tukey test, *p* < 0.05; 8-isoprostane, Dose main effect, *p* = 0.006, Dose 0 vs. 100 (†), Tukey test, *p* = 0.004; Macrophages, PM main effect, *p* = 0.018, TiO_2_ vs. SRM-1649 (†), Tukey test, *p* = 0.049, Dose main effect, *p* < 0.001, Dose 0 vs. 50, 100 or 250 (‡), Dose 50 vs. 250 (#), Tukey test, *p* < 0.05; Band cells, Dose main effect, *p* < 0.001, Dose 0 vs. 50 or 100 (†), Tukey test, *p* < 0.001; Lymphocytes, Not statistically significant. (DOCX 63 kb)

Additional file 6: Figure S5. Granulocyte Macrophage-Colony Stimulating Factor (GM-CSF) (A), interleukin (IL)-1α (B), IL-1β (C), IL-5 (D), IL-10 (E), Keratinocyte-derived Chemokine (KC) (F), Macrophage Inflammatory Protein (MIP)-1α (G), Regulated upon Activation Normal T cell Expressed and Secreted (RANTES) (H) and Tumor Necrosis Factor (TNF)-α (I) levels in bronchioalveolar lavage fluid of BALB/c mice exposed to particles by intratracheal instillation, at 24 h post-exposure. Values represent mean fold-effect (FE) ± standard error of the mean (*n* = 5). Two way ANOVA; GM-CSF, Dose main effect, *p* < 0.001, Dose 250 vs. 0 (†), Dose 250 vs. 50 (‡), Tukey test, *p* < 0.05; IL-1α, PM main effect, *p* < 0.001, CRI, EHC-2000 or SRM-1649 vs. DWR1 or TiO_2_ (†), Tukey test, *p* < 0.05, Dose main effect, *p* < 0.001, Dose 0 vs. 50, 100 or 250 (‡), Dose 50 vs. 100 (not shown), Dose 250 vs. 50 or 100 (not shown), Tukey test, *p* < 0.05; IL-β, PM × Dose interaction, *p* = 0.015, asterisks (*) represent effects significantly different from Dose 0 control, Tukey test, *p* < 0.05; IL-5, PM × Dose interaction, *p* = 0.007, asterisks (*) represent effects significantly different from Dose 0 control, Tukey test, *p* < 0.05; IL-10, not statistically significant; KC, PM × Dose interaction, *p* = 0.001, asterisks (*) represent effects significantly different from Dose 0 control, Tukey test, *p* < 0.05; MIP-1α, PM × Dose interaction, *p* < 0.001, asterisks (*) represent effects significantly different from Dose 0 control, Tukey test, *p* < 0.05; RANTES, Dose main effect, *p* < 0.001, Dose 0 vs. 100 or 250 (†), Dose 250 vs. 50 or 100 (‡), Tukey test, *p* < 0.05; TNF-α, PM main effect, *p* = 0.003, TiO_2_ vs. CRI, EHC-2000 or SRM-1649 (†), Tukey test, *p* < 0.05, Dose main effect, *p* < 0.001, Dose 0 vs. 50, 100 or 250 (‡), Dose 250 vs. 50 (#), Tukey test, *p* < 0.05. (DOCX 100 kb)
