# Supplementary material for: Development of an integrated approach for comparison of in vitro and in vivo responses to particulate matter
Source: Part Fibre Toxicol. 2016 Aug 12;13:41. doi: 10.1186/s12989-016-0152-6 (PMC4983025; doi:10.1186/s12989-016-0152-6)
Supplement: Supplementary file 1 — Elemental composition and size of the urban particulate matter and mineral particles. Table S2. Pearson correlations for cytotoxic potency and cytokine inductions in cell lines versus BALB/c mice exposed to particles. Table S3. Pearson correlations for the combined average in vitro (cell lines) and in vivo (BALB/c mice) particle potency estimates. (DOC 77 kb) [file 12989_2016_152_MOESM1_ESM.doc]

**TABLES**
