# Supplementary material for: Development of an integrated approach for comparison of in vitro and in vivo responses to particulate matter
Source: Part Fibre Toxicol. 2016 Aug 12;13:41. doi: 10.1186/s12989-016-0152-6 (PMC4983025; doi:10.1186/s12989-016-0152-6)
Supplement: Supplementary file 2 — Resazurin reduction by J774A.1 (A) and A549 (B) cells exposed to particles. Average fold-effect (FE) over control ± standard error values are shown (n = 5). ATP content of J774A.1 (C) and A549 (D) cells exposed to particles. (n = 3). Proliferation in J774A.1 (E) and A549 (F) cells exposed to particles. (n = 3). Two way ANOVA; Resazurin assay, J774A.1, PM main effect, p < 0.001, SRM-1648 vs. DWR1, EHC-98 or EHC-2000 (†), SRM-1649 vs. DWR1, EHC-93, EHC-98, EHC-2000, TiO2 or CRI (‡), Tukey test, p < 0.05, Dose main effect, p < 0.001, Doses 0, 10 or 20 vs. 40, 80 or 160 (#), Dose 40 vs. 80 or 160 (not shown), Dose 80 vs. 160 (not shown), Tukey test, p < 0.05; Resazurin assay, A549, PM × Dose interaction, p = 0.001, asterisks (*) represent effects significantly different from control, Tukey test, p < 0.05. ATP assay, J774A.1, PM main effect, p < 0.001, EHC-98 vs. SRM-1648, SRM-1649 or EHC-2000 (†), SRM-1649 vs. DWR1, TiO2 or EHC-93 (‡), Tukey test, p < 0.05, Dose main effect, p < 0.001, Dose 160 vs. all doses (#), Tukey test, p < 0.001; ATP assay, A549, PM main effect, p < 0.001, DWR1 vs. all particles (†), EHC-93 vs. SRM-1649, CRI or TiO2 (‡), Tukey test, p < 0.05, Dose main effect, p < 0.001, Dose 10 vs. 0, 40, 80 or 160 (not shown), Dose 20 vs. 80 (not shown), Dose 160 vs. 0, 20, 40 or 80 (#), Tukey test, p < 0.05. BrdU assay, J774A.1, PM main effect, p < 0.001, DWR1 or CRI vs. EHC-93, EHC-98, EHC-2000, SRM-1648, SRM-1649 or TiO2 (†), Tukey test, p < 0.05, Dose main effect, p < 0.001, Doses 0 or 10 vs. 20, 40, 80 or 160 (‡), Doses 20 or 40 vs. 80 or 160 (not shown), Dose 80 vs. 160 (not shown), Tukey test, p < 0.05; BrdU assay, A549, Dose main effect, p < 0.001, Dose 10 vs. 0, 20, 80 or 160 (†), Dose 40 vs. 80 or 160 (‡), Tukey test, p < 0.05. (DOCX 80 kb) [file 12989_2016_152_MOESM2_ESM.docx]

Figure S1

A B

C D

E F
