# Supplementary material for: Development of an integrated approach for comparison of in vitro and in vivo responses to particulate matter
Source: Part Fibre Toxicol. 2016 Aug 12;13:41. doi: 10.1186/s12989-016-0152-6 (PMC4983025; doi:10.1186/s12989-016-0152-6)
Supplement: Supplementary file 3 — Granulocyte-Macrophage Colony-Stimulating Factor (GM-CSF) (A), interleukin (IL)-1α (B), IL-β (C), IL-10 (D), Regulated upon Activation Normal T cell Expressed and Secreted (RANTES) (E) and Tumor Necrosis Factor (TNF)-α (F) levels in cell culture supernatants of J774A.1 cells exposed to particles for 24 h. Values are presented as mean fold-effect (FE) ± standard error (n = 3). Two way ANOVA; GM-CSF, PM × Dose interaction, p = 0.001, asterisks (*) represent effects significantly different from Dose 0 control, Tukey test, p < 0.05; IL-1α, PM × Dose interaction, p < 0.001, asterisks (*) represent effects significantly different from Dose 0 control, Tukey test, p < 0.05; IL-β, PM × Dose interaction, p < 0.001, asterisks (*) represent effects significantly different from Dose 0 control, Tukey test, p < 0.05; IL-10, PM × Dose interaction, p = 0.026, asterisks (*) represent effects significantly different from Dose 0 control, Tukey test, p < 0.05; RANTES, PM × Dose interaction, p < 0.001, asterisks (*) represent effects significantly different from Dose 0 control, Tukey test, p < 0.05; TNF-α, PM × Dose interaction, p < 0.001, asterisks (*) represent effects significantly different from Dose 0 control, Tukey test, p < 0.05. (DOCX 73 kb) [file 12989_2016_152_MOESM3_ESM.docx]

Figure S2

A B

C D

E F
