# Supplementary material for: Development of an integrated approach for comparison of in vitro and in vivo responses to particulate matter
Source: Part Fibre Toxicol. 2016 Aug 12;13:41. doi: 10.1186/s12989-016-0152-6 (PMC4983025; doi:10.1186/s12989-016-0152-6)
Supplement: Supplementary file 4 — Granulocyte-Macrophage Colony-Stimulating Factor (GM-CSF) (A), interleukin (IL)-1β (B), IL-8 (C), IL)-10 (D), Monocyte Chemoattractant Protein (MCP)-1 (E), Macrophage Inflammatory Protein (MIP)-1β (F) and Tumor Necrosis Factor (TNF)-α (G) levels in cell culture supernatants of A549 cells exposed to particles for 24 h. Values are presented as mean fold-effect (FE) ± standard error (n = 3). Two way ANOVA; GM-CSF, Dose main effect, p < 0.001, Dose 160 vs. 0, 10 or 40 (†), Dose 10 vs. 40 (not shown), Tukey test, p < 0.05; IL-1β, PM main effect, p = 0.004, TiO2 vs. DWR1 or EHC-93 (†), Tukey test, p < 0.05, Dose main effect, p < 0.001, Doses 40 or 160 vs. 0 or 10 (‡), Tukey test, p < 0.05; IL-8, PM × Dose interaction, p = 0.016, asterisks (*) represent effects significantly different from Dose 0 control, Tukey test, p < 0.05; IL-10, not statistically significant; MCP-1, PM × Dose interaction, p = 0.001, asterisks (*) represent effects significantly different from Dose 0 control, Tukey test, p < 0.05; MIP-1β, PM main effect, p = 0.014, TiO2 vs. EHC-93 (†), Tukey test, p < 0.05, Dose main effect, p = 0.023, Dose 160 vs. 10 (‡), Tukey test, p < 0.05; TNF-α, PM main effect, p < 0.001, TiO2 vs. DWR1, EHC-93 or EHC-2000 (†), CRI vs. DWR1 (‡), Tukey test, p < 0.05, Dose main effect, p < 0.001, Dose 0 vs. 10, 40 or 160 (#), Dose 10 vs. 40 (not shown), Dose 160 vs. 10 or 40 (not shown), Tukey test, p < 0.05. (DOCX 85 kb) [file 12989_2016_152_MOESM4_ESM.docx]

Figure S3

A B

C D

E F

G
