# Supplementary material for: Development of an integrated approach for comparison of in vitro and in vivo responses to particulate matter
Source: Part Fibre Toxicol. 2016 Aug 12;13:41. doi: 10.1186/s12989-016-0152-6 (PMC4983025; doi:10.1186/s12989-016-0152-6)
Supplement: Supplementary file 5 — Lactate dehydrogenase (LDH) levels in bronchioalveolar lavage (BAL) fluid (A), 8-isoprostane levels in blood plasma (B), Macrophage (C), Band cell (D) and lymphocyte (E) counts in BAL fluid from BALB/c mice exposed to particles by intratracheal instillation, at 24 h post-exposure. Values represent mean fold-effect (FE) ± standard error of the mean (n = 5). Two way ANOVA; LDH, PM × Dose interaction, p = 0.035, asterisks (*) represent effects significantly different from Dose 0 control, Tukey test, p < 0.05; 8-isoprostane, Dose main effect, p = 0.006, Dose 0 vs. 100 (†), Tukey test, p = 0.004; Macrophages, PM main effect, p = 0.018, TiO2 vs. SRM-1649 (†), Tukey test, p = 0.049, Dose main effect, p < 0.001, Dose 0 vs. 50, 100 or 250 (‡), Dose 50 vs. 250 (#), Tukey test, p < 0.05; Band cells, Dose main effect, p < 0.001, Dose 0 vs. 50 or 100 (†), Tukey test, p < 0.001; Lymphocytes, Not statistically significant. (DOCX 63 kb) [file 12989_2016_152_MOESM5_ESM.docx]

Figure S4

A B

C D

E
