# Supplementary material for: Development of an integrated approach for comparison of in vitro and in vivo responses to particulate matter
Source: Part Fibre Toxicol. 2016 Aug 12;13:41. doi: 10.1186/s12989-016-0152-6 (PMC4983025; doi:10.1186/s12989-016-0152-6)
Supplement: Supplementary file 6 — Granulocyte Macrophage-Colony Stimulating Factor (GM-CSF) (A), interleukin (IL)-1α (B), IL-1β (C), IL-5 (D), IL-10 (E), Keratinocyte-derived Chemokine (KC) (F), Macrophage Inflammatory Protein (MIP)-1α (G), Regulated upon Activation Normal T cell Expressed and Secreted (RANTES) (H) and Tumor Necrosis Factor (TNF)-α (I) levels in bronchioalveolar lavage fluid of BALB/c mice exposed to particles by intratracheal instillation, at 24 h post-exposure. Values represent mean fold-effect (FE) ± standard error of the mean (n = 5). Two way ANOVA; GM-CSF, Dose main effect, p < 0.001, Dose 250 vs. 0 (†), Dose 250 vs. 50 (‡), Tukey test, p < 0.05; IL-1α, PM main effect, p < 0.001, CRI, EHC-2000 or SRM-1649 vs. DWR1 or TiO2 (†), Tukey test, p < 0.05, Dose main effect, p < 0.001, Dose 0 vs. 50, 100 or 250 (‡), Dose 50 vs. 100 (not shown), Dose 250 vs. 50 or 100 (not shown), Tukey test, p < 0.05; IL-β, PM × Dose interaction, p = 0.015, asterisks (*) represent effects significantly different from Dose 0 control, Tukey test, p < 0.05; IL-5, PM × Dose interaction, p = 0.007, asterisks (*) represent effects significantly different from Dose 0 control, Tukey test, p < 0.05; IL-10, not statistically significant; KC, PM × Dose interaction, p = 0.001, asterisks (*) represent effects significantly different from Dose 0 control, Tukey test, p < 0.05; MIP-1α, PM × Dose interaction, p < 0.001, asterisks (*) represent effects significantly different from Dose 0 control, Tukey test, p < 0.05; RANTES, Dose main effect, p < 0.001, Dose 0 vs. 100 or 250 (†), Dose 250 vs. 50 or 100 (‡), Tukey test, p < 0.05; TNF-α, PM main effect, p = 0.003, TiO2 vs. CRI, EHC-2000 or SRM-1649 (†), Tukey test, p < 0.05, Dose main effect, p < 0.001, Dose 0 vs. 50, 100 or 250 (‡), Dose 250 vs. 50 (#), Tukey test, p < 0.05. (DOCX 100 kb) [file 12989_2016_152_MOESM6_ESM.docx]

Figure S5

A B

C D

E F

G H

I
